# N-acetylcysteine modulates neutrophil-driven immune and metabolic pathways in steatotic liver ischemia–reperfusion injury

**DOI:** 10.3389/fimmu.2026.1821592

**Published:** 2026-05-19

**Authors:** Jiman Kang, Digvijay Patil, Ryan Hackett, Yuki Cui, Kesha Oza, Abigail Rutkowski, Jedson R. Liggett, Henghong Li, Suman Ranjit, DongHyang Kwon, Bhaskar Kallakury, Chris Albanese, Gabriel E. Gondolesi, Udeme Ekong, Wanxing Cui, Khalid Khan, Thomas M. Fishbein, Alexander Kroemer

**Affiliations:** 1 MedStar Georgetown Transplant Institute, MedStar Georgetown University Hospital and the Center for Translational Transplant Medicine, Georgetown University Medical Center, Washington, DC, United States; 2 Department of Biochemistry and Molecular & Cellular Biology, Georgetown University, Washington, DC, United States; 3 Department of Surgery, Naval Medical Center Portsmouth, Portsmouth, VA, United States; 4 Department of Oncology, Lombardi Comprehensive Cancer Center, Georgetown University Medical Center, Washington, DC, United States; 5 Department of Pathology, MedStar Georgetown University Hospital, Washington, DC, United States

**Keywords:** hepatic steatosis, immunometabolism, ischemia-reperfusion injury, lipid remodeling, N-acetylcysteine

## Abstract

Hepatic ischemia-reperfusion injury (IRI) is a complex event influenced by interconnected immune and metabolic processes. Steatotic livers are especially sensitive to IRI, but the crosstalk between innate inflammatory responses and lipid metabolic dysregulation in this context is not well understood. Using transcriptomic profiling in a murine high-fat diet (HFD) model, we assessed immune and metabolic responses to hepatic IRI and examined the effects of N-acetylcysteine (NAC). In steatotic livers, IRI induced the upregulation of inflammatory mediators, including TLR/NF-κB-associated genes (*Tlr2*, *Tlr4*, *Irf1*) and neutrophil-associated genes (*S100a8*, *S100a9, Lcn2*), accompanied by the downregulation of lipid and cholesterol metabolism-related genes, including *Cyp7a1*, *Cyp8b1*, *Cyp27a1*, and *Hmgcr*. NAC supplementation attenuated inflammatory gene expression and restored key lipid biosynthetic regulators. We then performed targeted lipidomic analysis to determine whether NAC-mediated transcriptional changes were reflected at the lipid level and observed a significant increase in total phosphatidylcholine and sphingomyelin in steatotic livers following IRI. Finally, to assess the contribution of innate immune cells to hepatic IRI, we quantified neutrophils and macrophages in HFD+NAC IRI and HFD IRI livers. We found that NAC supplementation reduced hepatic neutrophil accumulation and markedly decreased LCN2 expression following IRI.

## 1 Introduction

Ischemia-reperfusion injury (IRI) is a key contributor to early allograft dysfunction, allograft rejection, and reduced graft survival after liver transplantation and surgical liver resection (1, 2). The combination of ischemia and abrupt reperfusion lead to the disruption of hepatocellular metabolic homeostasis and the amplification of inflammatory responses within the graft (1, 2). Steatotic livers are more vulnerable to IRI, with greater immune cell infiltration, more severe hepatocellular damage, and poorer survival compared with non-steatotic livers (3, 4).

The pathogenesis of IRI occurs in separate but overlapping phases. The initial phase of IRI is characterized by mitochondrial dysfunction, causing ATP depletion, massive reactive oxygen species (ROS) generation, and endothelial injury (5). The release of ROS leads to activation of resident hepatic macrophages (Kupffer cells) by damage-associated molecular patterns (DAMPs) (6). During the late phase of IRI, a robust inflammatory cascade is driven by the release of proinflammatory cytokines including tumor necrosis factor–α (TNF-α) and interleukin-1β (IL-1β) (5, 7). These cytokines further lead to extensive neutrophil activation, exacerbating hepatocyte injury and ultimately promoting necrotic cell death and tissue damage (8).

Lipocalin-2 (LCN2), also known as neutrophil gelatinase-associated lipocalin, is implicated in inflammation, cancer progression, and metabolic regulation (9). LCN2 expression is rapidly upregulated in response to organ injury, including damage to the brain, lung, and kidney, and is widely used as a sensitive biomarker of liver damage (9, 10). Beyond its role as an inflammatory marker, recent evidences suggest that LCN2 plays a key role in regulating hepatic lipid metabolism and inflammatory signaling (11, 12). Notably, *Lcn2* deficiency leads to dysregulation of mitochondrial phospholipid biosynthesis and mitochondrial-associated inflammation in brown adipose tissue in male mice, highlighting a broader role for *Lcn2* in metabolic and inflammatory homeostasis (11).

Because oxidative stress and inflammatory signaling are key drivers of hepatic IRI, cellular reduction-oxidation targeted therapeutic approaches have gained increasing attention. N-acetylcysteine (NAC), a thiol-containing synthetic compound, serves as a potent antioxidant agent with the capacity to scavenge ROS and act as a precursor for glutathione (GSH) synthesis (13). In experimental models of liver injury, NAC has been shown to attenuate oxidative stress, inhibiting the ROS-mediated apoptosis pathway, and modulate inflammatory signaling pathways (14, 15). We previously demonstrated that NAC attenuates ischemia-reperfusion injury in steatotic livers by reducing CD45^+^CD3^+^CD1d^+^ Type1 Natural Killer T-cells, implicating immune modulation as a key component of its protective effects (16). Clinically, NAC is widely used for the treatment of acetaminophen-induced acute liver failure (17) and has demonstrated hepatoprotective benefits in non–acetaminophen acute liver failure and other forms of acute liver injury (17). Although NAC is well known for its antioxidant activity, its immunometabolic effects during hepatic IRI, particularly in steatotic livers, have yet to be fully understood.

Given the antioxidant and anti-inflammatory roles of NAC in attenuating markers of inflammation and oxidative stress in hepatic injury, we hypothesized that NAC protects steatotic livers from IRI by selectively inhibiting immune activation and metabolic remodeling pathways that drive tissue injury. To test this hypothesis, we integrated histologic, immunologic, transcriptomic, and lipidomic analyses to define NAC-modulated injury networks during hepatic IRI.

## 2 Materials and methods

### 2.1 Experimental animals and diets

All animal procedures were approved by the Georgetown University Institutional Animal Care and Use Committee (IACUC; protocol #2016-1351). Male C57BL/6 mice were obtained at six weeks of age from Jackson Laboratory, Bar Harbor, ME. The mice were housed in the Division of Comparative Medicine at Georgetown University Medical Center under a standard 12-hour light/dark cycle. C57BL/6 mice were maintained on either a normal chow diet (ND, 5K52, LabDiet, St. Louis MO; 4.09 kcal/gram, 13.4% kJ/fat), a 60% high-fat diet (HFD, 58Y1, TestDiet, St. Louis MO; 5.10 kcal/gram, 60% kJ/fat), or HFD supplemented with 1%(w/v) N-acetylcysteine (HFD+NAC), as previously described (16).

At 18–20 weeks of age, mice underwent 70% warm partial hepatic ischemia using an atraumatic vascular clamp for 45- or 90-minutes, followed by reperfusion for either 24 hours or 7 days (**Supplementary Figure S1A**). Two ischemia durations were used in this study. A 45-minute warm partial hepatic ischemia model was used for mechanistic analyses, including RNA sequencing and targeted lipidomics, consistent with our previously published method (16, 18) to reliably induce reproducible hepatic injury without excessive mortality. Whereas, a more severe 90-minute ischemia model was used to assess survival and long-term histologic injury. Sham controls underwent laparotomy without vascular clamping. Livers were harvested at the assigned reperfusion time points.

### 2.2 Histology and immunohistochemistry

Formalin-fixed, paraffin-embedded liver tissues were sectioned at 5µm thickness and stained with hematoxylin and eosin (H&E) and Masson’s trichrome (MTS). Following tissue processing, H&E staining was performed using an automated stainer (Autostainer XL; Leica Biosystems), as previously described (16). All slides were reviewed in a blinded manner by an experienced hepatopathologist.

Liver injury was graded using the modified Suzuki scoring system, which evaluates hepatocellular necrosis, sinusoidal congestion, and hepatocellular ballooning/vacuolization. Each parameter is scored semi-quantitatively from 0 to 4 and summed to generate a final Suzuki score that reflects the severity of ischemia–reperfusion injury. In addition, steatohepatitis was assessed using the modified Nonalcoholic Steatohepatitis Clinical Research Network (NASH-CRN) scoring system, which independently evaluates macrovesicular (macro) steatosis, microvesicular (micro) steatosis, lobular inflammation, hepatocellular ballooning, and the stage and extent of fibrosis. Both the modified Suzuki and NASH-CRN scoring systems have been applied in human and murine models, as previously described (19, 20).

### 2.3 Targeted lipidomic profiling

Targeted lipidomic profiling was performed as previously described (16). Briefly, snap-frozen liver tissue was dissolved in ice-cold isopropyl alcohol (IPA) containing class-specific internal standards (IS), followed by freeze–thaw cycles (37 °C water bath or 90 seconds). After centrifugation, clarified extracts were analyzed by LC–MS/MS using an Xbridge amide column (3.5 µm, 4.6 × 100 mm, Phenomenex) coupled to a 5500 QTRAP triple quadrupole mass spectrometer (SCIEX) operating in multiple reaction monitoring (MRM) modes. To ensure accurate quantification, Peak areas were normalized to internal standards, and metabolite ratios were calculated from the normalized peak intensities. For statistical analysis, the bar graph data are expressed as mean ± standard error of the mean (SEM), and Mann-Whitney U tests were performed for comparisons.

### 2.4 Serum alanine aminotransferase measurement

Serum alanine aminotransferase (ALT) levels were measured using mouse ALT assay kits from Abcam according to the manufacturer’s instructions. For early reperfusion time points (24 and 48 hours), when ALT levels were expected to be high, the Abcam ALT Mouse Assay Kit (ab105134) was used in a kinetic format. Absorbance was measured at 450nm over a 60-minute period using a microplate reader.

### 2.5 Isolation of hepatic leukocytes and flow cytometry

Hepatic leukocytes were isolated as previously described (18). Briefly, liver tissues from HFD Sham, HFD IRI, and HFD+NAC IRI mice were harvested and placed in RPMI-1640 medium (Gibco). Tissues were mechanically dissociated and passed through a 70μm cell strainer (Fisher Scientific). Leukocyte fractions were isolated using a Percoll (Cytiva) density gradient. Following centrifugation at 1000 × g for 20 min at 25 °C without brake, the upper layer containing hepatocytes and debris was discarded. The leukocyte layer was collected, washed, and resuspended in 1× PBS (Gibco).

Liver leukocytes were stained with fluorochrome-conjugated antibodies; PerCP Cy5.5-conjugated anti-F4/80 (BioLegend), APC-conjugated anti-Ly6G (BioLegend), PE/Cyanine7-conjugated anti-MHCII (BioLegend), Alexa Fluor^®^ 700-conjugated CD45 (BioLegend), Brilliant Violet 510™-conjugated anti-CD11b (BioLegend), Brilliant Violet 650™-conjugated anti-CD11c (BioLegend). Dead cells were excluded using Zombie NIR™ live/dead viability dye (BioLegend). Data were acquired using a BD FACSAria III cytometer (BD Biosciences) at the Flow Cytometry & Cell Sorting Shared Resource (FCSR).

Following surface marker staining, cells were fixed and permeabilized using fixation/permeabilization buffer (eBioscience), and intracellular LCN2 was detected using an Alexa Fluor 488-conjugated anti-mouse LCN2 (NGAL) antibody (clone M047A10, BioLegend). Samples with viability ≤60% were excluded from all analyses. Fluorescence-minus-one (FMO) controls were used to define background fluorescence for both surface markers and intracellular LCN2 staining.

### 2.6 RNA-seq and analysis

For the RNA-seq experiment, mouse liver specimens from sham or hepatic IRI with 45 minutes of 70% partial clamping were harvested 24 hours post-surgery. Liver tissue specimens were stored in Allprotect Tissue Reagent (Cat. no. 76405, Qiagen) and lysed with 1.0 mm Zirconia/Silica beads (BioSpec Products) using a FastPrep 24 Tissue Homogenizer (MP Biomedical). Total RNA was then extracted from the mouse liver tissue with the RNeasy Mini kit, including the RNase-free DNase set (Cat. no. 74004, Qiagen). RNA samples with RNA integrity score RIN > 6 were used for sequencing.

Sequencing library preparation, sequencing, and bioinformatics analysis were performed at the Novogene Sequencing facility (Novogene, Inc., Sacramento, CA). Sequencing libraries were generated using NEBNext^®^ Ultra II RNA Library Prep Kit for Illumina (NEB, USA) to produce paired-end, 150-bp, poly(A) enriched cDNA libraries. Libraries were sequenced on an Illumina NovaSeq 6000 platform to obtain approximately 30 million paired-end 150-bp reads per sample (~6Gb per sample) with ≥85% of sequenced bases achieving a Phred quality score ≥30 (Q30). Sequencing reads were mapped to Mus Musculus (GRCm39/mm39) reference genome using HISAT2 (21), and featureCounts (22) were used to assign mapped reads to genes. Each group has at least three biological replicates. Raw gene counts were used as input to the DESeq2 (23) for differential gene expression analysis across groups, with adjusted p-values < 0.05 considered statistically significant.

### 2.7 Statistical analysis

All statistical analyses (Mann–Whitney U test statistics and Multiple t-tests) were performed using GraphPad Prism (version 10; GraphPad Software, San Diego, CA), all graphs show mean ± SEM unless stated otherwise. Statistically significant differences were set as a p-value <0.05.

## 3 Results

### 3.1 NAC attenuates IRI-induced histologic injury in steatotic livers

**Figure 1:**
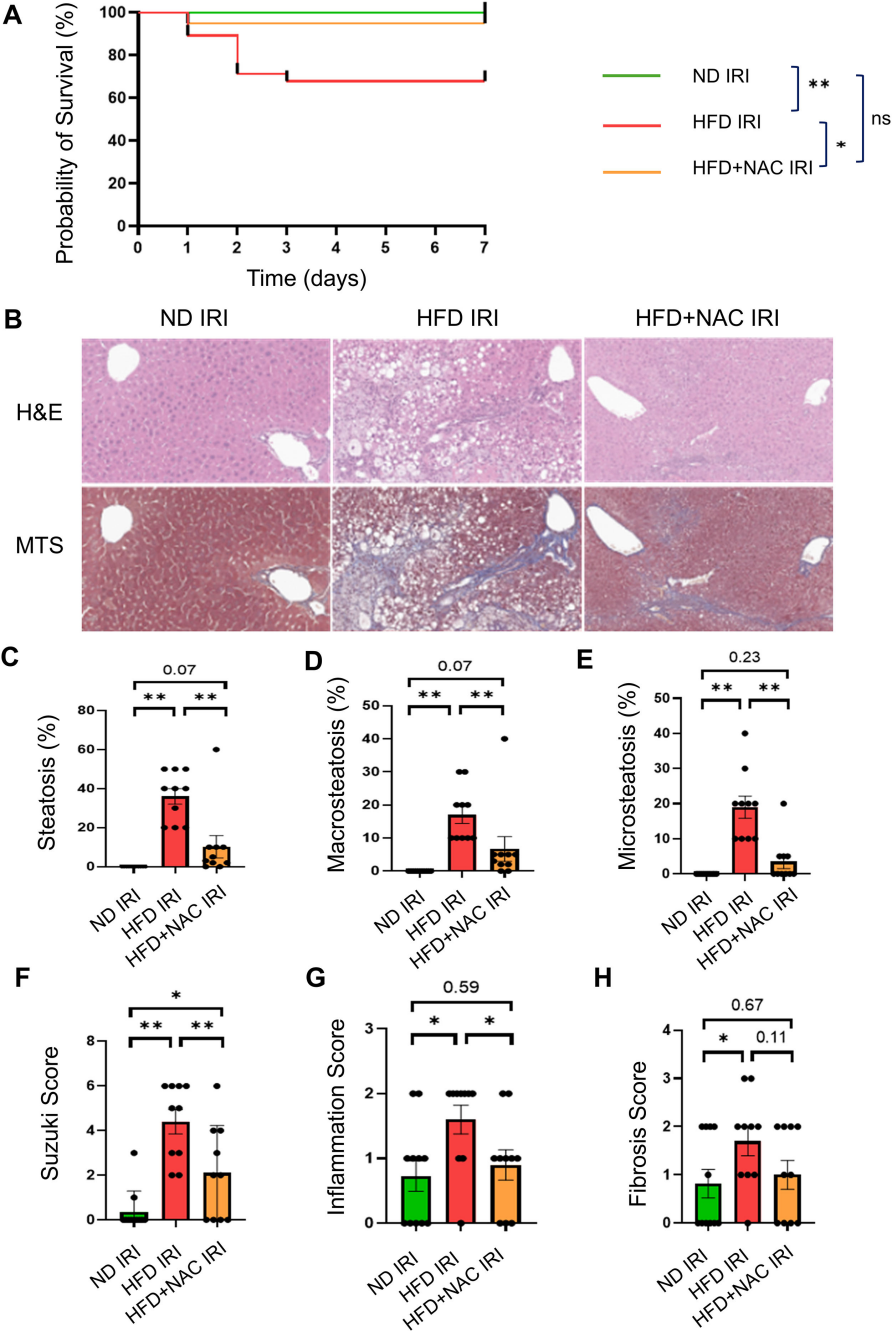
NAC improves survival and reduces steatosis, inflammation and liver injury in steatotic IRI. **(A)** Kaplan-Meier survival curves. **(B)** Representative H&E and Masson’s trichrome (MTS) staining of liver sections from normal diet (ND), high-fat diet (HFD), and high-fat diet supplemented with N-acetylcysteine (HFD+NAC) following 90 minutes of warm ischemia and 7 days of reperfusion. **(C–E)** Quantification of hepatic steatosis, including **(C)** total steatosis, **(D)** macrosteatosis, and **(E)** microsteatosis. **(F–H)** Histopathologic assessment of IRI-induced liver damage, including **(F)** Suzuki score (necrosis, sinusoidal congestion, and hepatocyte ballooning), **(G)** inflammation score (portal and lobular inflammatory infiltrates), and **(H)** fibrosis score based on MTS staining. Statistical significance was determined using one-way ANOVA with Tukey’s *post hoc* test **(B–H)** and survival differences were assessed using the log-rank (Mantel–Cox) test. The number of mice analyzed ranged from 22 (ND IRI) to 28 (HFD IRI) to 20 (HFD+NAC IRI). Exact *p* values are indicated in the image. **p < 0.01, *p < 0.05, ns, not significant.

We first used a 90-minute hepatic IRI model in steatotic livers to characterize survival and tissue-level injury and to evaluate long-term NAC-derived protective effects during prolonged ischemia. Kaplan–Meier survival analysis demonstrated that HFD mice exhibited significantly higher 7-day mortality following 90-minute IRI compared with both ND IRI (p<0.01) and HFD+NAC IRI (p<0.05) (**Figure 1A**). Next, we evaluated hepatocellular injury by measuring circulating alanine aminotransferase (ALT) levels. At 24 hours after IRI, ALT values were similar between HFD and HFD+NAC mice in the sham groups (**Supplementary Figure S2B**). However, at the 48-hour time point following IRI, HFD+NAC mice displayed significantly lower ALT levels than HFD mice, indicating reduced hepatocellular injury with NAC supplementation.

To further characterize tissue-level injury and fibrosis, livers were harvested 7 days after 90-minute warm ischemia for histopathologic analysis. H&E and Masson’s trichrome staining (**Figure 1B**) revealed marked injury in HFD livers, including prominent macrovesicular (macro) and microvesicular (micro) steatosis, hepatocellular injury, increased inflammation, and fibrosis (**Figures 1C–H**). Semi-quantitative scoring by pathological evaluation of H&E and MTS staining confirmed that HFD IRI mice exhibited significantly higher total steatosis, macrosteatosis, microsteatosis, Suzuki injury scores, inflammation scores, and fibrosis compared with ND IRI controls. NAC supplementation significantly reduced total steatosis, macrosteatosis, microsteatosis, Suzuki injury scores, and inflammation relative to HFD IRI. In contrast, fibrosis scores were not significantly reduced by NAC at 7 days post-IRI. Together, these findings indicate that NAC attenuates steatotic liver injury and inflammatory responses following IRI.

### 3.2 Ischemia–reperfusion injury results in distinct transcriptional alterations in steatotic livers

**Figure 2:**
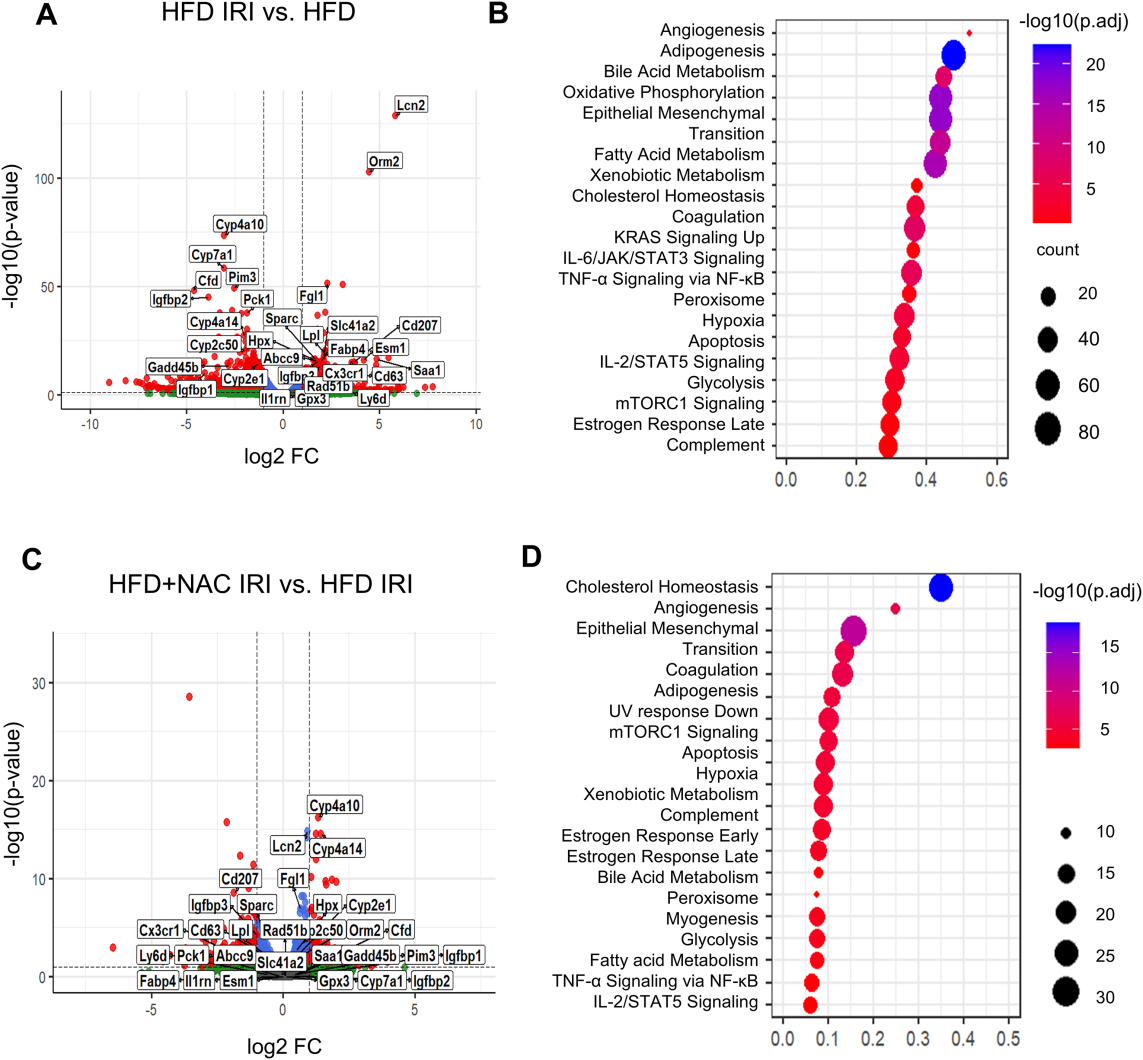
NAC attenuates IRI-induced transcriptional alterations in steatotic livers. **(A, C)** Volcano plots showing differentially expressed genes (DEGs). **(A)** HFD IRI vs. sham HFD controls (HFD). **(C)** HFD+NAC IRI vs. HFD IRI. **(B, D)** Pathway enrichment analysis using MSigDB Hallmark gene sets (24). Bubble size represents the number of genes enriched in each pathway; color indicates–log_10_(adjusted p-value). DEGs were defined using padj < 0.05.

To define transcriptional changes associated with hepatic IRI, we analyzed bulk RNA-seq data from livers of HFD mice following IRI and compared them with sham HFD controls (**Figure 2A**). Differential expression analysis showed increased expression of inflammatory and stress-associated genes, including *Lcn2*, *Orm2*, *Ly6d*, and *Saa1*. Critically, we found a markedly lower transcript abundance of lipid and bile acid metabolism-associated genes, such as *Cyp7a1*, *Cyp8b1*, and *Cyp27a1*, in comparison to HFD IRI. Pathway enrichment analysis identified activation of inflammatory and stress-related pathways, including IL-6/JAK/STAT3 signaling, TNF-α signaling via NF-κB, and complement activation, as well as alterations in metabolic pathways related to oxidative phosphorylation, fatty acid metabolism, and cholesterol homeostasis (**Figure 2B**).

To assess the impact of NAC on steatotic livers subjected to IRI, we compared livers from HFD+NAC IRI and HFD IRI groups. With NAC treatment, IRI-induced gene expression changes were partially reversed, with increased expression of lipid metabolism–related genes (*Cyp4a10*, *Cyp4a14*, *Fdps*) and lower expression of inflammatory genes such as *Lcn2*, *Orm2*, and *Cd63* (**Figure 2C**). Enrichment analysis identified pathways related to cholesterol homeostasis, angiogenesis, and epithelial remodeling in NAC-treated livers (**Figure 2D**).

To determine whether NAC alters hepatic responses under baseline conditions independent of IRI, we compared HFD and HFD+NAC groups (**Supplementary Figure S3**). NAC treatment under baseline conditions reduced the expression of selected inflammatory and redox-related genes, including *Lcn2*, *Orm2*, and *Gstm3*. Notably, these genes were strongly induced during IRI (HFD IRI vs HFD) and subsequently attenuated by NAC treatment (HFD+NAC IRI vs HFD IRI). These findings suggest that NAC modifies baseline hepatic inflammatory and redox status but also attenuates additional gene induction triggered by IRI.

### 3.3 NAC alters injury-associated transcriptional responses in steatotic liver IRI

**Figure 3:**
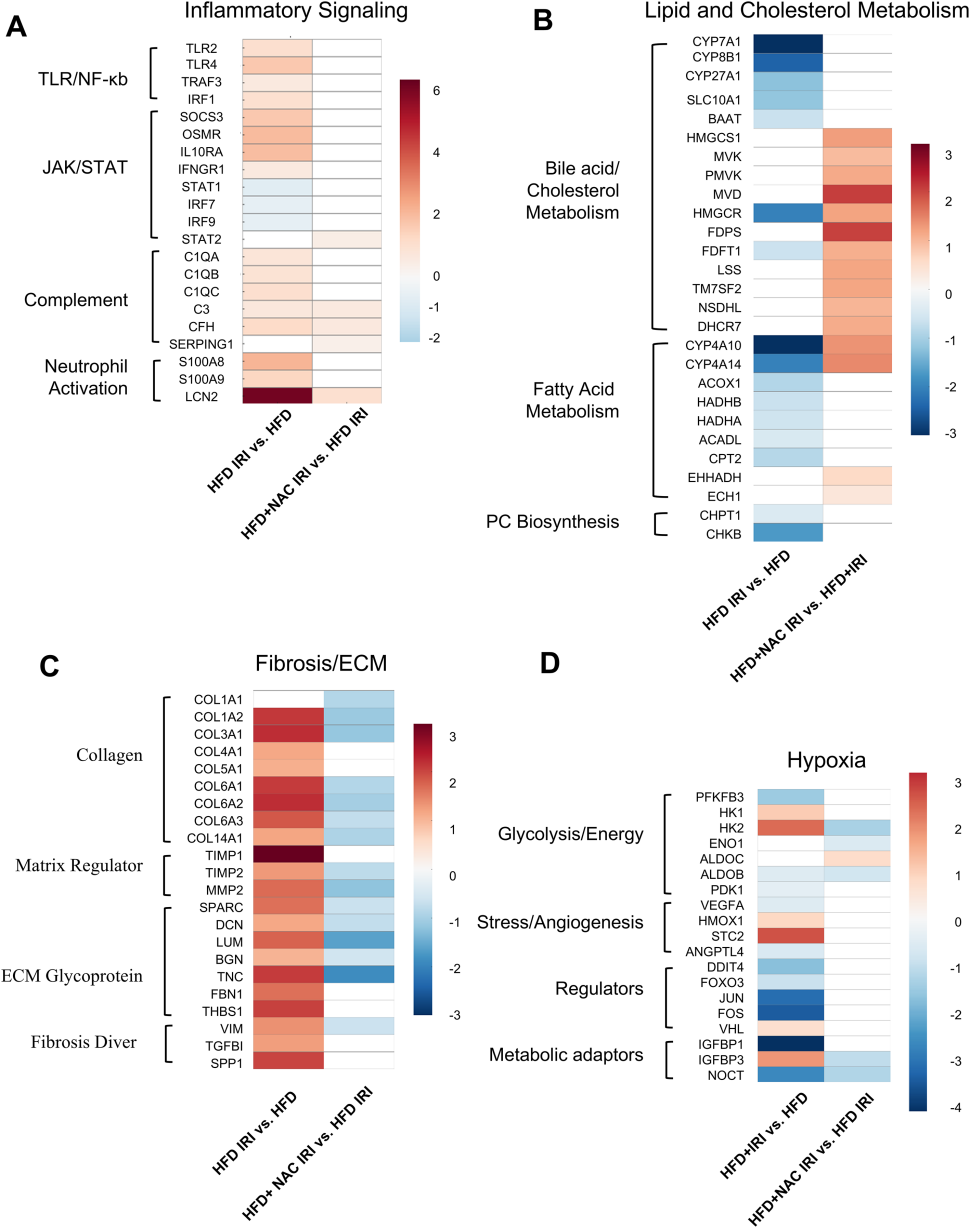
NAC selectively alters transcriptional genes involved in inflammatory, lipid/cholesterol, fibrosis/ECM and hypoxia metabolic in steatotic IRI. **(A)** Inflammatory signaling. HFD IRI upregulated innate immune and inflammatory pathways, including TLR/NF-κB, JAK/STAT, complement, and neutrophil activation signatures. NAC treatment suppressed these proinflammatory genes, including *LCN2* and *S100a8/a9*. **(B)** Lipid and cholesterol metabolism. HFD IRI markedly downregulated bile acid metabolism (*Cyp7a1*, *Slc10a1*), fatty acid oxidation (*Cyp4a10*, *Acox1*, *Cpt2*), and PC biosynthesis (*Chpt1*, *Chkb*). **(C)** Fibrosis/ECM remodeling. HFD IRI induced a fibrotic program with upregulation of collagens (*Col1a2*, *Col3a1*, *Col6a1/2/3*), matrix regulators (*Timp1*, *Mmp2*), ECM glycoproteins (*Sparc*, *Lum*, *Bgn*, *Dcn*, *Thbs1*, *Fbn1*), and fibrosis drivers (*Vim*, *Tgfbi*, *Spp1*). NAC treatment suppressed this profibrotic transcriptional signature. **(D)** Hypoxia response. White cells indicate no significant change (padj > 0.05). Data shown as log_2_ fold change.

We examined key pathway genes related to IRI, including those involved in inflammation, metabolism, fibrosis, and hypoxia, to assess the effects of NAC on IRI-induced transcriptional changes (**Figure 3**). IRI in steatotic livers markedly upregulated multiple inflammatory mediators (**Figure 3A**), including TLR/NF-κB–associated genes (*Tlr2*, *Tlr4*, *Irf1*) and the cytokine-responsive feedback regulator *Socs3*, as well as neutrophil-associated genes (*S100a8*, *S100a9*, *Lcn2*). NAC supplementation (HFD+NAC IRI) attenuated this inflammatory transcriptional response-associated genes no longer reaching statistical significance relative to HFD IRI.

Critically, we found a markedly lower transcript abundance of genes involved in lipid and cholesterol metabolism (**Figure 3B**), including *Cyp7a1*, *Cyp8b1*, *Cyp27a1*, and *Hmgcr* in HFD IRI livers relative to HFD livers. Interestingly, HFD+NAC IRI livers exhibited reprogramming of lipid metabolic pathways (**Figure 3B**), characterized by the upregulation of cholesterol biosynthesis (*Hmgcs1*, *Mvd*, *Fdps*) genes and fatty-acid oxidation genes (*Cyp4a10*, *Cyp4a14*). However, the transcript levels of key phosphatidylcholine biosynthesis genes (*Chpt1*, *Chk*) remained unchanged in HFD+NAC IRI livers relative to HFD IRI.

The epithelial-to-mesenchymal transition (EMT) is a well-characterized driver of liver fibrosis and tumor metastasis (25). EMT and matrix metalloproteinase (MMP) activation have been reported to be exacerbated in steatotic livers following IRI compared with lean livers (26). In our model, HFD IRI livers significantly induced transcriptional programs associated with fibrosis and extracellular matrix (ECM) remodeling (**Figure 3C**). Genes encoding collagen isoforms, matrix regulators, and ECM glycoproteins were strongly upregulated, reflecting activation of fibrogenic pathways. NAC treatment reversed the IRI-induced upregulation of several EMT-associated genes, including those related to ECM remodeling (*Col1a2*, *Mmp2*, *Bgn*, *Col6a2*, *Col5a2*) and fibrosis (*Tnc*, *Vim*). Together, these findings indicate that NAC mitigates the fibrotic and matrix-remodeling transcriptional responses triggered by IRI in steatotic livers. In addition to fibrotic remodeling, IRI triggered a pronounced hypoxia-associated transcriptional response in steatotic livers, which was only partially attenuated by NAC (**Figure 3D**).

### 3.4 Shared and NAC-resistant gene reveals selective reprogramming of injury networks

To further understand the NAC-mediated modulation, we analyzed genes commonly altered across the HFD IRI vs. HFD and HFD+NAC IRI vs. HFD IRI comparisons (**Supplementary Figure S4**). We selected the shared 388 genes and visualized the molecular pathways (**Supplementary Figure S4A**). We identified key enrichment of pathways related to cholesterol homeostasis, EMT, angiogenesis, and adipogenesis, indicating coordinated regulation of metabolic and structural remodeling programs. Heatmap visualization revealed distinct gene expression patterns across the HFD IRI vs. HFD and HFD+NAC IRI vs. HFD IRI comparisons. In particular, relative to HFD controls, HFD IRI livers showed significant upregulation of genes associated with EMT, cell death, and hypoxia (**Supplementary Figure S4**). In contrast, these injury-associated transcriptional programs were attenuated in HFD+NAC IRI livers compared with HFD IRI alone. However, genes involved in cholesterol homeostasis and adipogenesis were relatively upregulated in HFD+NAC IRI livers relative to HFD IRI (**Supplementary Figure S4C**). Collectively, these findings indicate that NAC exerts protective effects by selectively reprogramming overlapping transcriptional networks that are dysregulated during IRI in steatotic livers.

In parallel, we examined gene sets uniquely altered in HFD IRI that were not reversed by NAC treatment (**Supplementary Figure S5**). Venn diagram analysis revealed a non-overlapping gene set between the HFD IRI vs. HFD and HFD+NAC IRI vs. HFD IRI comparisons (**Supplementary Figure S5A**). We focused on the 2,890 genes uniquely altered in HFD IRI, representing a set of NAC-resistant genes. Pathway enrichment analysis of this gene set identified processes related to oxidative phosphorylation, xenobiotic metabolism, fatty acid metabolism, KRAS signaling, TNF-α/NF-κB signaling, and EMT. Finally, we examined genes uniquely altered by NAC treatment relative to HFD IRI (**Supplementary Figure S6**). Venn diagram analysis revealed a set of non-overlapping genes between the two groups (**Supplementary Figure S6A**). The 299 NAC-responsive genes that were not shared with HFD+IRI were enriched for pathways, including cholesterol homeostasis, complement and coagulation cascades, hypoxia, xenobiotic metabolism, and mTORC1 signaling. Collectively, these findings suggest that NAC selectively regulates metabolic and stress-response pathways without globally suppressing the entire transcriptional response to IRI.

### 3.5 Global lipidomic profiling reveals NAC-induced enhancement of glycerophospholipid abundance

**Figure 4:**
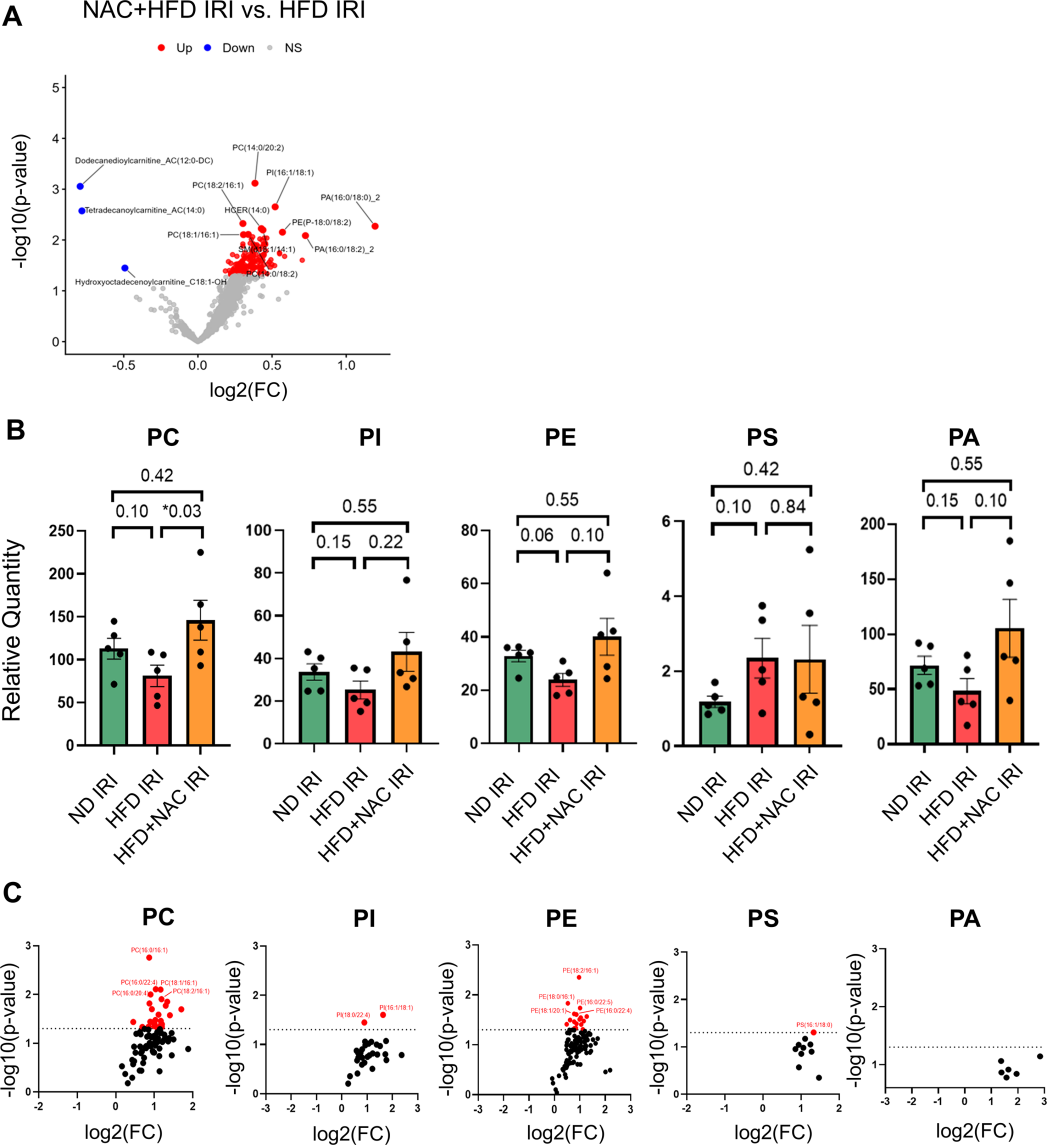
Lipidomic analysis of glycerophospholipids in steatotic liver IRI. **(A)** Volcano plots of specific lipid species comparing HFD+NAC IRI vs HFD IRI groups. **(B)** Quantification of liver glycerophospholipids, including phosphatidylcholine (PC), phosphatidylinositol (PI), phosphatidylethanolamine (PE), phosphatidylserine (PS), and phosphatidic acid (PA) after partial warm hepatic ischemia and 24-hour reperfusion period. **(C)** Volcano plots of specific PC, PI, PE, PS, and PA species comparing HFD+NAC IRI vs HFD IRI groups. For bar graphs, Mann Whitney U tests were used. For volcano plots, Student’s t-tests were used. Each point represents an individual lipid species plotted by log_2_ fold change and –log_10_(p value). Red dots indicate significantly increased lipid species in the HFD+NAC IRI group (p < 0.05), as defined by the dashed horizontal threshold line. Blue indicates downregulation and red upregulation of lipid species. N = 5 mice per group.

Given the transcriptomic evidence of dysregulated lipid and phosphatidylcholine biosynthesis following IRI (**Figure 3B**), we further tested our hypothesis by performing a lipidomic analysis of hepatic tissue using targeted lipidomic profiling by LC–MS/MS (**Supplementary Table S1**). Global lipidomic analysis revealed a marked shift in lipid abundance with NAC treatment (HFD+NAC IRI) compared with HFD IRI. Volcano plots demonstrated a strong directional bias toward lipid species increased in HFD+NAC IRI livers compared with HFD IRI controls, with both substantial fold changes and statistical significance (**Figure 4A**).

Among the 781 lipid species quantified, 77 were increased in HFD+NAC IRI livers compared with HFD IRI, with nearly two-thirds of these changes involving glycerophospholipid species (**Figure 4B**). These changes were concordant with the transcriptomic findings and suggest that NAC promotes lipid remodeling and preservation of membrane-associated phospholipids disrupted by ischemic stress. Previous studies have shown that hepatic steatosis exacerbates IRI-induced depletion of key phospholipids, particularly phosphatidylcholines (PCs), which are critical for membrane fluidity and organelle integrity (27). Consistent with this, total PC levels were reduced in HFD IRI livers compared with ND IRI controls and were significantly restored following NAC treatment (**Figures 4B, C**). Other glycerophospholipids, including phosphatidylethanolamine (PE) and phosphatidylserine (PS), did not show significant differences between HFD IRI and HFD+NAC IRI livers.

### 3.6 NAC selectively preserves sphingomyelin in steatotic liver IRI

**Figure 5:**
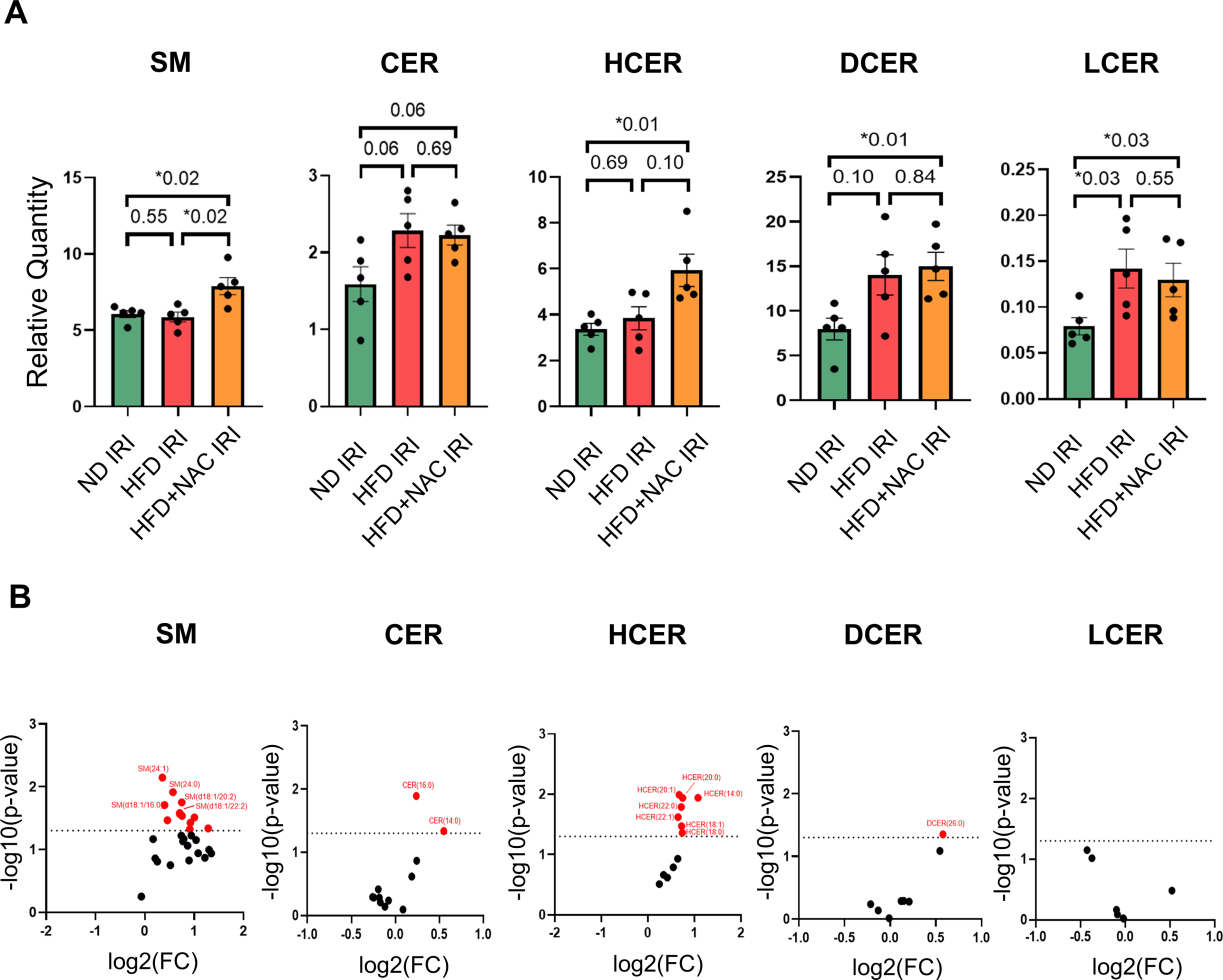
Lipidomic analysis of sphingomyelins and ceramides. **(A)** Quantification of liver; sphingomyelin (SM), ceramide (CER), hexosylceramide (HCER), dihydroceramide (DCER), and lactosylceramide (LCER) after partial warm hepatic ischemia and 24-hour reperfusion period. **(B)** Volcano plots of specific SM, CER, HCER, DCER, and LCER species comparing HFD+NAC IRI vs HFD IRI groups. N = 5 mice per group. For bar graphs, Mann–Whitney U test was used. For volcano plots, Student’s t-test was used. “*” indicates statistical significance (p < 0.05).

Since sphingomyelins and ceramides are essential structural components of cell membranes and play important roles in stress signaling, membrane structure, cell death, and signal transduction, we next quantified hepatic sphingomyelin and ceramide species following IRI. Of the 77 lipid species increased with NAC, two were ceramide-class lipids, and twelve were sphingomyelin species (**Figure 5**). Sphingomyelin was unchanged between ND IRI and HFD IRI, but NAC supplementation significantly increased sphingomyelin relative to HFD IRI (**Figure 5A**). Compared with ND IRI, total ceramide levels showed an upward trend in both HFD IRI and HFD+NAC IRI (both p≈0.06). Compared with ND IRI, lactosylceramide levels were increased in both HFD IRI and HFD+NAC IRI livers. In contrast, hexosylceramide and dihydroceramide were selectively elevated in HFD+NAC IRI livers (**Figure 5**). NAC did not significantly alter these ceramide subclasses compared with HFD IRI. Additional lipid classes—including lysophospholipids, free fatty acids, acylglycerols, acylcarnitines, and cholesterol species—showed minimal or nonsignificant changes across groups (**Supplementary Figures S7**-**S10**). Collectively, these data demonstrate that NAC reprograms injury-responsive metabolic pathways at both the transcriptional and lipidomic levels, selectively enhancing membrane-stabilizing glycerophospholipids and sphingolipids.

### 3.7 NAC attenuates neutrophil recruitment and LCN2 induction in steatotic liver IRI

**Figure 6:**
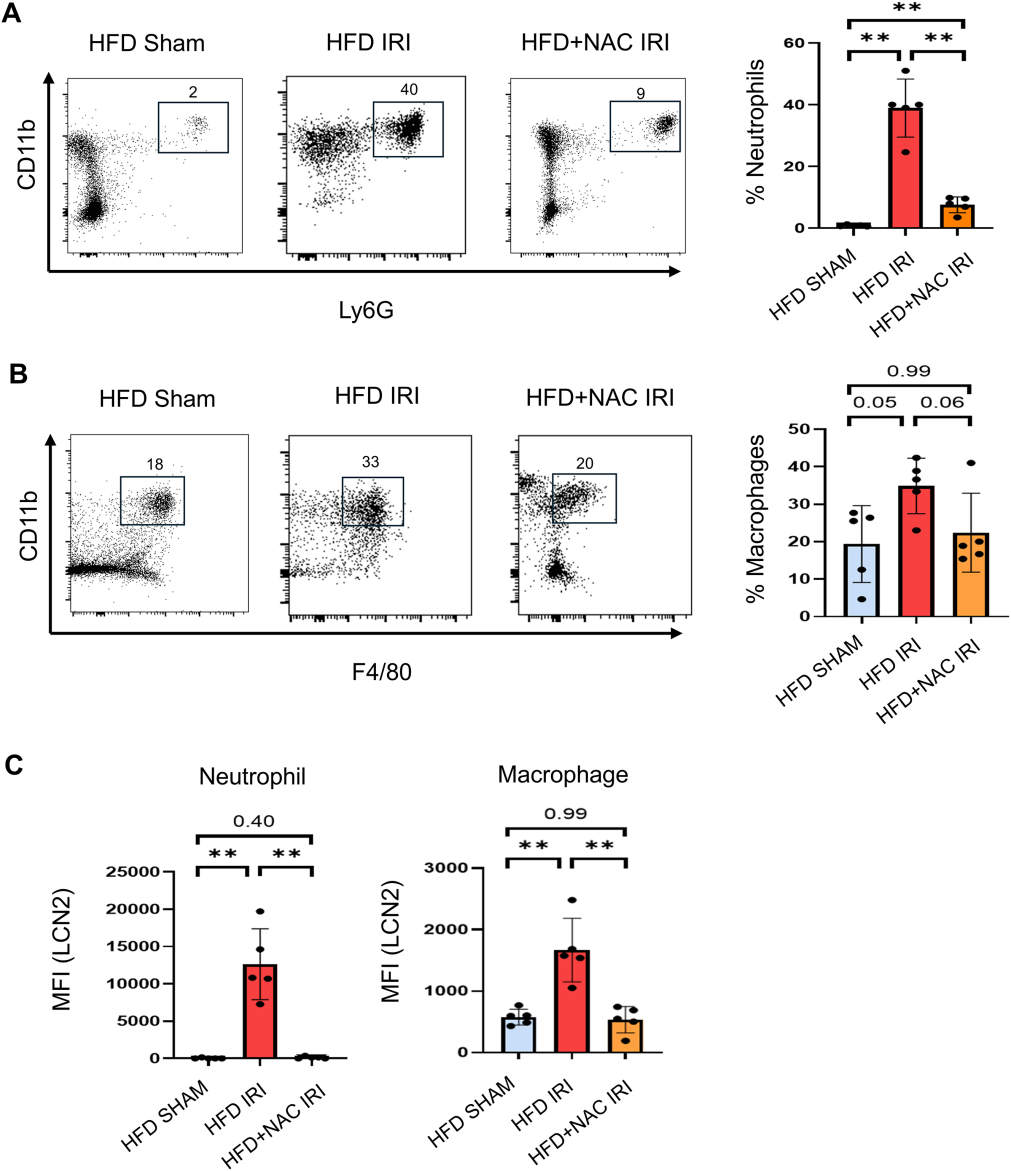
NAC reduces hepatic neutrophil accumulation and suppresses LCN2 expression following steatotic IRI. **(A)** Representative flow cytometry plots and quantification of hepatic neutrophils (CD45^+^CD11b^+^Ly6G^+^) in HFD Sham, HFD IRI, HFD+NAC IRI. Percentages indicate the frequency of neutrophils within CD45^+^ cells. **(B)** Representative flow cytometry plots and quantification of hepatic macrophages (CD45^+^CD11b^+^F4/80^+^) across experimental groups. **(C)** Mean fluorescence intensity (MFI) of LCN2 expression in hepatic neutrophils and macrophages. Data are shown as mean ± SD. Each dot represents an individual mouse. Mann–Whitney U test was used; *p* values are indicated in the figure. **P <0.01.

To validate the transcriptomic evidence that NAC attenuates injury-associated inflammatory signaling in steatotic hepatic IRI (**Figure 3A**), we next quantified hepatic innate immune cell populations, including neutrophils, macrophages, and dendritic cells in liver tissue by flow cytometry. The representative flow cytometric gating strategy is shown in **Supplementary Figure S11**. Neutrophils were defined as CD45^+^CD11b^+^Ly6G^+^ cells, macrophages as CD45^+^CD11b^+^Ly6G^-^F4/80^+^ cells, and dendritic cells as CD45^+^CD11b^+^MHCII^+^ cells (**Supplementary Figure S11**). Compared with HFD sham controls, HFD IRI mice exhibited a significant increase in Ly6G^+^CD11b^+^ neutrophils. NAC treatment significantly reduced neutrophil accumulation, with neutrophil frequencies returning toward sham levels (**Figure 6A**). In contrast, F4/80^+^CD11b^+^ macrophages exhibited only a modest increase following IRI, which was partially attenuated by NAC supplementation. The frequency of CD11b^+^MHCII^+^ dendritic cells did not differ significantly among groups, suggesting that NAC does not broadly suppress innate immune cell populations (**Supplementary Figure S11**).

Based on the marked upregulation of neutrophil-associated inflammatory genes observed in our transcriptomic analysis of HFD IRI livers, we next assessed LCN2 expression as a marker of inflammatory activation within hepatic neutrophils and macrophages. Neutrophils from HFD IRI livers exhibited a significant increase in LCN2 mean fluorescence intensity (MFI) compared with sham controls, whereas NAC treatment markedly reduced LCN2 expression (**Figure 6C**). Similarly, LCN2 expression in macrophages was significantly elevated following IRI and was attenuated by NAC treatment toward baseline levels. Together, these findings demonstrate that NAC reduces neutrophil accumulation and suppresses LCN2 expression in both neutrophils and macrophages following hepatic IRI.

## 4 Discussion

IRI is an unavoidable consequence of liver-related surgery and transplantation. Currently, there are no established highly specific biomarkers for the diagnosis or prognosis of hepatic IRI, and no approved pharmacologic therapies to prevent or treat this condition. NAC, a glutathione precursor, exhibits remarkable antioxidant activity and improves liver function during ischemic injury by reducing ROS and inhibiting ROS-mediated cell death. However, little is known about how NAC regulates inflammatory and metabolic signaling, particularly immune–metabolic crosstalk in steatotic livers.

First, we characterized survival and tissue-level injury by employing a 90-minute steatotic liver IRI model. Although a 45-minute IRI model is highly clinically relevant and well-suited for delineating early mechanisms of hepatocellular injury and inflammatory pathway activation (16), this duration did not produce measurable differences in mortality (**Supplementary Figure S2A**) or fibrotic remodeling between ND and HFD mice. We therefore extended the ischemia duration to 90 minutes to enable assessment of survival and of longer-term histologic, immunologic, and metabolic effects of oral NAC treatment.

Within the 7-day reperfusion period (**Figure 1**), HFD mice develop significantly more fibrosis than ND controls. In contrast, fibrosis scores in HFD+NAC IRI livers were not significantly different from those in HFD IRI, although a downward trend was observed. Given that fibrotic remodeling represents a delayed and a longer-term structural response to hepatic injury, often persisting even after resolution of acute hepatocellular damage (28), it is possible that fibrosis scores were not significantly reduced by NAC in steatotic livers at the 7-day time point. Longer reperfusion intervals may therefore be required to detect potential antifibrotic effects of NAC. These findings suggest that NAC has translational potential to enhance the resilience of steatotic marginal grafts to IRI.

We analyzed the biological pathways for the HFD IRI vs. HFD and HFD+NAC IRI vs. HFD IRI, and the intersection of the comparison groups to elucidate key pathways of NAC modulation. Our RNA-seq analyses demonstrate that NAC exerts selective pathway reprogramming rather than global suppression of steatotic IRI–associated injury responses. HFD livers showed broad transcriptional remodeling mainly involved in inflammatory cytokine signaling, hypoxia responses, EMT, and oxidative stress pathways compared to HFD IRI livers. NAC significantly attenuated a subset of these immune–metabolic networks, particularly those associated with neutrophil activation, inflammatory signaling, and cell death. Consistent with our findings, a recent study reported that N-acetyl cysteine amide (NACA), a lipophilic derivative of NAC, attenuated inflammatory cell infiltration and reduced caspase-3 immunoreactivity—a key enzyme in programmed cell death—in a rat renal ischemia–reperfusion injury model (29). Importantly, not all IRI-induced pathways were reversed by NAC. For example, genes induced by steatotic IRI but not significantly altered by NAC treatment included pathways related to oxidative phosphorylation among the top 10 pathways (**Supplementary Figure S4**).

A key finding of this study is that NAC treatment modulates hepatic phospholipid remodeling during steatotic IRI. HFD IRI livers showed a trend toward reduced abundance of phosphatidylcholines, a membrane-stabilizing component, relative to ND IRI, consistent with NAFLD/MAFLD-associated phospholipid dysregulation that sensitizes hepatocytes to metabolic stress (27, 30, 31). NAC restored phosphatidylcholine abundance, aligning transcriptomic and lipidomic data to support a model in which NAC improves hepatocyte resilience by maintaining membrane homeostasis and limiting susceptibility of hepatocyte membranes to ischemia-associated oxidative stress.

Importantly, although NAC significantly reduced histologic steatosis–including both macrovesicular and microvesicular lipid droplet accumulation (**Figures 1C–E**)–RNA-seq analyses revealed upregulation of cholesterol metabolic pathways and preservation of phospholipid remodeling programs. Together with targeted lipidomic data, these findings indicate enhanced lipid turnover and membrane remodeling rather than increased intracellular lipid storage, consistent with improved lipid flux during recovery from ischemic stress.

Consistent with our finding of reduced PC in HFD IRI livers, a recent study reported a significant decrease in total PC following IRI in the steatotic livers, supporting the idea that disruption of PC homeostasis is a feature of IRI (27). Although the Kennedy pathway is the primary route for PC synthesis, we did not observe significant transcriptional alterations in core Kennedy pathway enzymes. The restoration of phosphatidylcholine levels may reflect post-transcriptional regulation or altered availability of CDP-choline, as total diacylglycerol levels were not significantly changed (**Supplementary Figure S7C**). It is therefore possible that improved lipid flux and membrane remodeling—rather than direct activation of *de novo* PC synthesis via the Kennedy pathway—contribute to PC preservation following NAC treatment. Adequate cholesterol availability appears to be a critical determinant of hepatic adaptive responses after injury. It has been suggested that activation of *de novo* cholesterol biosynthesis supports hepatocyte metabolic remodeling and liver regeneration (32). More recently, TCA cycle intermediates have been shown to feed into cholesterol synthesis pathways during these regenerative processes (33). Consistent with these observations, the upregulation of cholesterol metabolism–associated genes in HFD+NAC IRI livers may help stabilize membrane lipid and enhance hepatocyte resilience.

Phospholipids are susceptible to (per)oxidation under oxidative stress, generating oxidized phospholipid (OxPL) epitopes that function as endogenous DAMPs and are recognized by innate immune receptors such as scavenger receptors and toll-like receptors (TLRs) (34, 35). In hepatic IRI, OxPLs accumulate during injury and serve as endogenous ligands for neutrophil TLR dependent pathways, inducing activation and recruitment of these cells (36, 37). Several studies have reported that inhibition of TLR4/NF-κB signaling dampens oxidative stress and inflammatory responses and attenuates hepatic IRI (38, 39). Consistent with this framework, we observed robust upregulation of TLR/NF-κB-associated genes (*Tlr2*, *Tlr4*, *Irf1*) and neutrophil-associated molecules (*S100a8*, *S100a9*, *Lcn2*) in HFD IRI livers compared to HFD livers, which was attenuated by NAC treatment. Given that NAC can reduce ROS and lipid peroxidation (40), it is plausible that NAC may limit OxPL formation, thereby attenuating OxPL-dependent neutrophil activation and recruitment.

Sphingolipids play important roles in regulating intracellular signaling cascades and membrane integrity during hepatic IRI (41, 42). A recent rat study examining the effects of NAC on sphingolipid pathways under ND- and HFD- conditions demonstrated that NAC regulates protein levels of serine palmitoyltransferase long chain base subunit 1 (SPTLC1) and SPTLC2. (43), suggesting that NAC can modulate *de novo* sphingolipid synthesis. SPTLC1 and SPTLC2 are required for serine palmitoyltransferase (SPT) activity, the rate-limiting step in sphingolipid biosynthesis (44). Our data showed HFD+NAC IRI livers exhibited increased sphingomyelin abundance relative to HFD IRI. In contrast, other sphingolipid species, including ceramide, hexosylceramide, dihydroceramide, and lactosylceramide, did not show significant changes (**Figure 5**). Together, these data suggest that NAC treatment under steatotic liver IRI conditions preferentially preserves membrane-stabilizing sphingomyelin rather than broadly affecting sphingolipid metabolism.

Although NAC is widely recognized for its anti-inflammatory effects, it has remained unclear whether NAC specifically limits neutrophil recruitment and activation during steatotic hepatic IRI, or instead broadly suppresses myeloid cell responses. To address this question, we performed flow cytometry. HFD IRI livers showed a significant increase in hepatic neutrophil (CD45^+^CD11b^+^Ly6G^+^) accumulation, which was significantly attenuated by NAC supplementation. However, the macrophage (CD45^+^CD11b^+^Ly6G^-^F4/80^+^) population in HFD+NAC IRI livers showed more modest changes and was not significantly reduced compared to HFD IRI livers, suggesting that NAC preferentially targets neutrophil-driven inflammatory injury. Previous studies have demonstrated that LCN2 can be induced by multiple stress and inflammatory pathways, including TLR/NF-κB and cytokine-driven JAK/STAT signaling (45, 46). In our dataset, NAC attenuated TLR/NF-κB–associated inflammatory programs and reduced LCN2 expression, particularly in neutrophils. Together, these findings suggest that NAC may reduce oxidative stress/lipid peroxidation in HFD IRI livers, thereby dampening TLR/NF-κB–mediated inflammatory signaling and limiting neutrophil activation and LCN2 induction.

Although reduction of oxidative stress is likely a central mechanism of NAC activity, emerging evidence suggests that NAC may also influence inflammatory signaling pathways beyond direct ROS scavenging. NAC has been reported to influence key signaling cascades such as NF-κB (47–49) and JAK/STAT pathways (50), which regulate cytokine production and immune activation. In our dataset, the coordinated attenuation of inflammatory gene expression and neutrophil-associated responses suggests that NAC-mediated protection likely reflects both antioxidant effects and broader modulation of immune-metabolic signaling networks rather than an exclusively ROS-dependent mechanism.

We observed increased phosphatidylcholine and sphingomyelin abundance in HFD+NAC IRI livers compared to HFD IRI livers, accompanied by reduced neutrophil activation and LCN2 expression. Lipid raft organization is known to regulate neutrophil chemotaxis, adhesion, and signaling (37a, 51), and disruptions in phosphatidylcholine metabolism have been linked to enhanced neutrophil migration in inflammatory disease (13). Together, these findings suggest that NAC may limit excessive reperfusion injury by stabilizing membrane lipid architecture and dampening neutrophil-driven inflammation. These coordinated transcriptomic and lipidomic changes are consistent with previous findings linking transcriptional regulation of lipid metabolism to membrane remodeling during liver injury (52).

In conclusion, our study presents novel insight into the influence of NAC on immune–metabolic stress responses in steatotic livers, effectively dampening neutrophil-driven inflammation and restoring lipid membrane homeostasis. As steatotic donor livers are increasingly used because of rising obesity and the persistent shortage of donor organs, transplantation has adopted strategies such as normothermic machine perfusion (NMP) to improve graft outcomes. Our findings suggest that NAC supplementation, administered systemically or during ex vivo perfusion, may help enhance graft resilience by stabilizing immune–metabolic responses and limiting IRI. Although NAC was administered orally in this study, additional studies using clinically relevant delivery approaches are needed. Future studies using neutrophil depletion strategies or LCN2-targeted approaches will be important to directly determine the contribution of the neutrophil–LCN2 axis to NAC-mediated protection in steatotic liver IRI.

## Data Availability

The datasets presented in this study are available from the corresponding author upon request.

## Supplementary material

Supplementary Figure S1 Experimental design and representative gross liver images from the steatotic IRI model following IRI. **(A)** Schematic of the experimental design for hepatic IRI. Following clamp release, mice were allocated to 24-hour or 7-day reperfusion groups, and livers were harvested at the indicated time points. **(B–D)** Representative gross hepatic image at 7 days post-IRI in mice fed **(B)** ND, **(C)** HFD, or **(D)** HFD+NAC. Images depict the left hepatic lobe.

Supplementary Figure S2 **(A)** Kaplan–Meier survival curves of warm IRI in ND and HFD mice. **(B)** Serum alanine aminotransferase (ALT) levels in ND IRI, HFD IRI, and HFD+NAC IRI mice at sham, 24 hours, and 48 hours following 90 minutes of IRI. Statistical comparisons were performed using Student t-test. **p < 0.01; ns=not significant.

Supplementary Figure S3 Baseline effects of NAC and modulation of inflammatory and redox-related gene expression during IRI. **(A)** Heatmap showing selected inflammatory, immune signaling, and redox-related genes across three comparisons: HFD+NAC vs HFD, HFD IRI vs HFD, and HFD+NAC IRI vs HFD IRI. Data shown as log_2_ fold change. **(B)** The summary table highlights representative genes (*Lcn2, Orm2, and Gstm3*).

Supplementary Figure S4 NAC selectively modulates IRI-responsive genes. **(A)** A Venn diagram showing the overlap of differentially expressed genes (DEGs) between HFD IRI vs. HFD and HFD+NAC IRI vs. HFD IRI. A total of 388 shared DEGs represent genes induced by IRI that were also modified by NAC treatment. A dot plot shows enriched Hallmark pathways among the 388 shared genes. **(B–E)** Heatmaps visualizing log_2_ fold changes of representative genes involved in **(B)** EMT, **(C)** Cholesterol Homeostasis and Adipogenesis, **(D)** Cell Death, and **(E)** Hypoxia. Gene expression values are shown as log_2_ fold changes relative to respective control comparisons.

Supplementary Figure S5 **(A)** A Venn diagram comparing DEGs from HFD IRI vs HFD and HFD+NAC IRI vs HFD IRI. The large unshared gene set comprising 2890 DEGs uniquely altered in steatotic IRI (i.e., not reversed by NAC) was analyzed for pathway enrichment. The dot plot shows the top enriched pathways, ranked by gene ratio and –log_10_(adjusted p-value). **(B)** List of representative genes contributing to each of the top enriched pathways identified in Panel **(A)**.

Supplementary Figure S6 **(A)** A Venn diagram showing differentially expressed genes (DEGs) from HFD IRI vs HFD and HFD+NAC IRI vs HFD IRI comparisons. The non-overlapping NAC-responsive gene set (299 genes) was subjected to pathway enrichment analysis. A dot plot displays the top 10 significantly enriched pathways, ranked by gene ratio and –log_10_(adjusted p-value). **(B)** List of representative genes contributing to each of the top enriched pathways identified in Panel **(A)**.

Supplementary Figure S7 Lipidomic analysis of lysophospholipids. Quantification of liver lysophospholipids **(A)** lysophosphatidylcholine (LPC), **(B)** lysophosphatidylinositol (LPI), **(C)** Lysophosphatidylethanolamine (LPE), and **(D)** Lysophosphatidic acid after partial warm hepatic ischemia and 24-hour reperfusion period. Volcano plots of specific **(E)** LPC, **(F)** LPI, **(G)** LPE, and **(H)** LPA species comparing HFD+NAC vs HFD groups. N = 5 mice per group. For bar graphs, Mann–Whitney U tests were used. For volcano plots, Student t-test was used.

Supplementary Figure S8 Lipidomic analysis of free fatty acids and acylglycerols. Quantification of liver **(A)** free fatty acid (FFA), **(B)** monoacylglycerol (MAG), **(C)** diacylglycerol (DAG), and **(D)** triacylglycerol (TAG) after partial warm hepatic ischemia and 24-hour reperfusion period. Volcano plots of specific **(E)** FFA, **(F)** MAG, **(G)** DAG, and **(H)** TAG species comparing HFD+NAC IRI vs HFD IRI groups. N = 5 mice per group. For bar graphs, Mann–Whitney U test was used. For volcano plots, Student t-test was used.

Supplementary Figure S9 Lipidomic analysis of mitochondrial lipid transport species. Quantification of liver **(A)** acylcarnitine (AC) after partial warm hepatic ischemia and 24-hour reperfusion period. Volcano plots of specific **(B)** AC species comparing HFD+NAC IRI vs HFD IRI groups. N = 5 mice per group. For bar graphs, Mann–Whitney U test was used. For volcano plots, Student t-test was used.

Supplementary Figure S10 Lipidomic analysis of conjugated and unconjugated cholesterol species. Quantification of liver **(A)** Cholesterol and **(B)** Cholesteryl Ester (CE) after partial warm hepatic ischemia and 24-hour reperfusion period. Volcano plots of specific **(C)** Cholesteryl Ester species comparing HFD+NAC IRI vs HFD IRI groups. N = 5 mice per group. For bar graphs, Mann Whitney U test was used. For volcano plots, Student t-test was used.

Supplementary Figure S11 Flow cytometric gating strategy and dendritic cell frequencies in steatotic livers subjected to IRI **(A)** Representative flow cytometric gating strategy used to identify hepatic immune cell populations. Neutrophils were defined as CD45^+^CD11b^+^Ly6G^+^ cells, macrophages as CD45^+^CD11b^+^F4/80 CD45^+^ cells, and dendritic cells as CD45^+^CD11b^+^MHCII^+^ cells. **(B)** Quantification of hepatic dendritic cell frequencies across HFD Sham, HFD IRI, and HFD+NAC IRI groups. No significant differences were observed among groups. Each dot represents an individual mouse. Statistical significance was assessed using Mann–Whitney U; *p* values are indicated in the figure.

Supplementary Table S1 The complete list of lipid species.
