# Correction: N-acetylcysteine modulates neutrophil-driven immune and metabolic pathways in steatotic liver ischemia–reperfusion injury

**DOI:** 10.3389/fimmu.2026.1886718

**Published:** 2026-07-07

**Authors:** Jiman Kang, Digvijay Patil, Ryan Hackett, Yuki Cui, Kesha Oza, Abigail Rutkowski, Jedson R. Liggett, Henghong Li, Suman Ranjit, DongHyang Kwon, Bhaskar Kallakury, Chris Albanese, Gabriel E. Gondolesi, Udeme Ekong, Wanxing Cui, Khalid Khan, Thomas M. Fishbein, Alexander Kroemer

**Affiliations:** 1 MedStar Georgetown Transplant Institute, MedStar Georgetown University Hospital and the Center for Translational Transplant Medicine, Georgetown University Medical Center, Washington, DC, United States; 2 Department of Biochemistry and Molecular & Cellular Biology, Georgetown University, Washington, DC, United States; 3 Department of Surgery, Naval Medical Center Portsmouth, Portsmouth, VA, United States; 4 Department of Oncology, Lombardi Comprehensive Cancer Center, Georgetown University Medical Center, Washington, DC, United States; 5 Department of Pathology, MedStar Georgetown University Hospital, Washington, DC, United States

**Keywords:** hepatic steatosis, immunometabolism, ischemia-reperfusion injury, lipid remodeling, N-acetylcysteine

Firstly, an incorrect **Funding** statement was provided. The correct **Funding** statement reads:

“The authors declare that financial support was received for this research and/or publication. Funding was provided by the Children’s Rare Disease Organization Inc. (CRDO) (AK, JK, KK).”

Secondly, the **Data availability** statement was erroneously given as:

“The datasets presented in this study can be found in online repositories. The names of the repository/repositories and accession number(s) can be found in the article/Supplementary Material.”

The correct **Data availability** statement is:

“The datasets presented in this study are available from the corresponding author upon request.”

And finally, an outdated version of **Supplementary File 1** was published with the original article. The file has now been replaced with the revised complete version.

The authors apologize for these errors and state that this does not change the scientific conclusions of the article in any way. The original article has been updated.

