# Supplementary material for: N-acetylcysteine modulates neutrophil-driven immune and metabolic pathways in steatotic liver ischemia–reperfusion injury
Source: Front Immunol. 2026 May 19;17:1821592. doi: 10.3389/fimmu.2026.1821592 (PMC13226007; doi:10.3389/fimmu.2026.1821592)
Supplement: Supplementary Figure S1 — Experimental design and representative gross liver images from the steatotic IRI model following IRI. (A) Schematic of the experimental design for hepatic IRI. Following clamp release, mice were allocated to 24-hour or 7-day reperfusion groups, and livers were harvested at the indicated time points. (B–D) Representative gross hepatic image at 7 days post-IRI in mice fed (B) ND, (C) HFD, or (D) HFD+NAC. Images depict the left hepatic lobe. [file DataSheet1.pdf]

Supplementary Figure S1

A

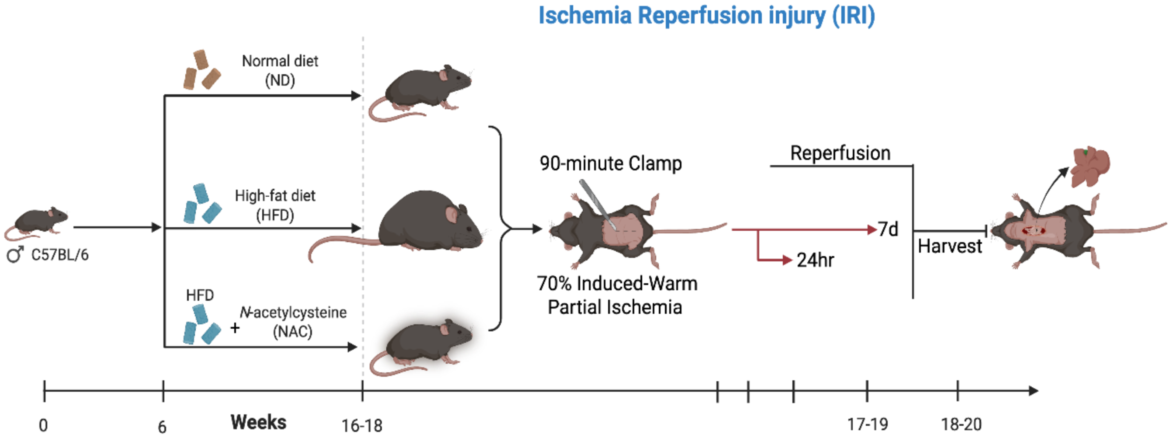

B **C** **D**

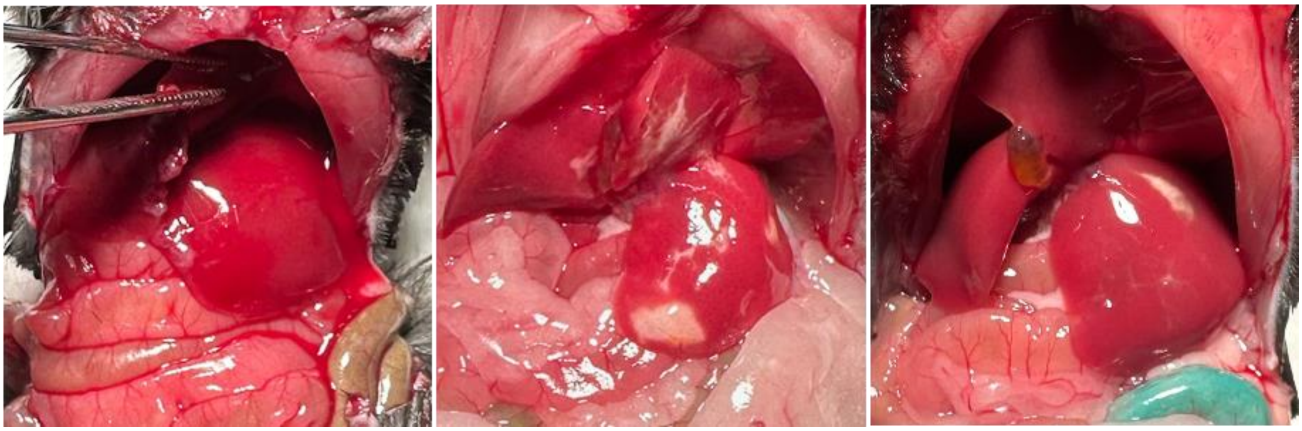

Supplementary Figure S2

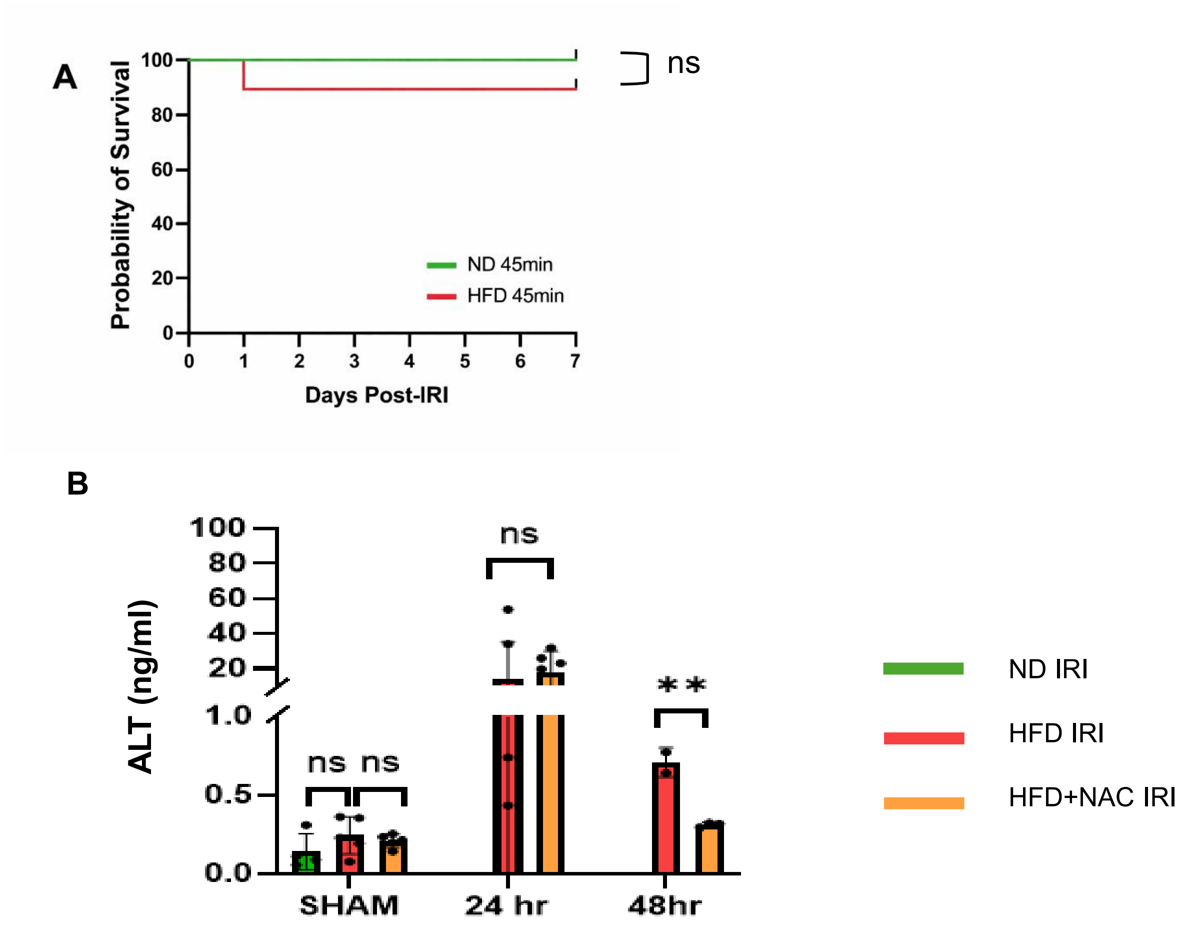

Supplementary Figure S3

A

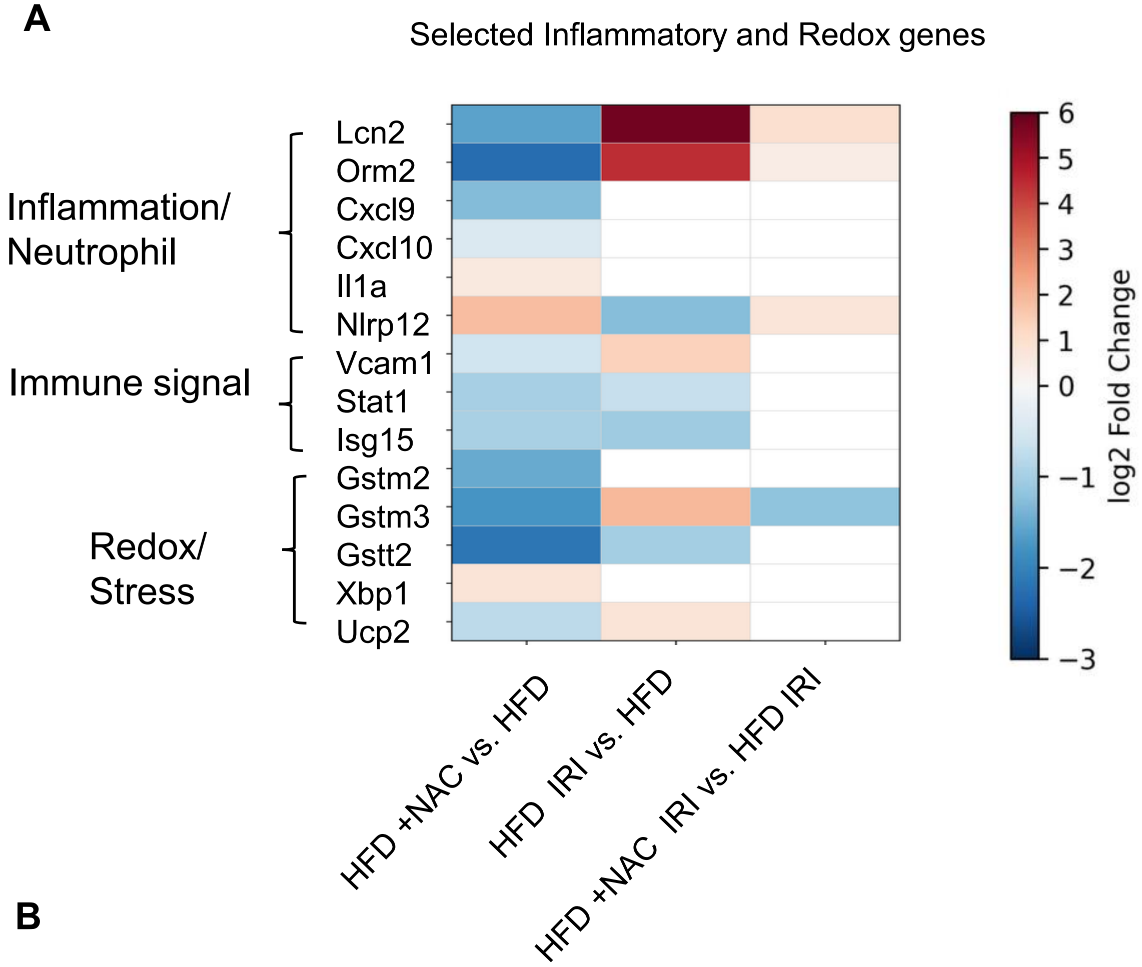

B

Gene	Baseline NAC	IRI effect	NAC during IRI
**Lcn2**	↓	↑	↓
**Orm2**	↓	↑	↓
**Gstm3**	↓	↑	↓

Supplementary Figure S4

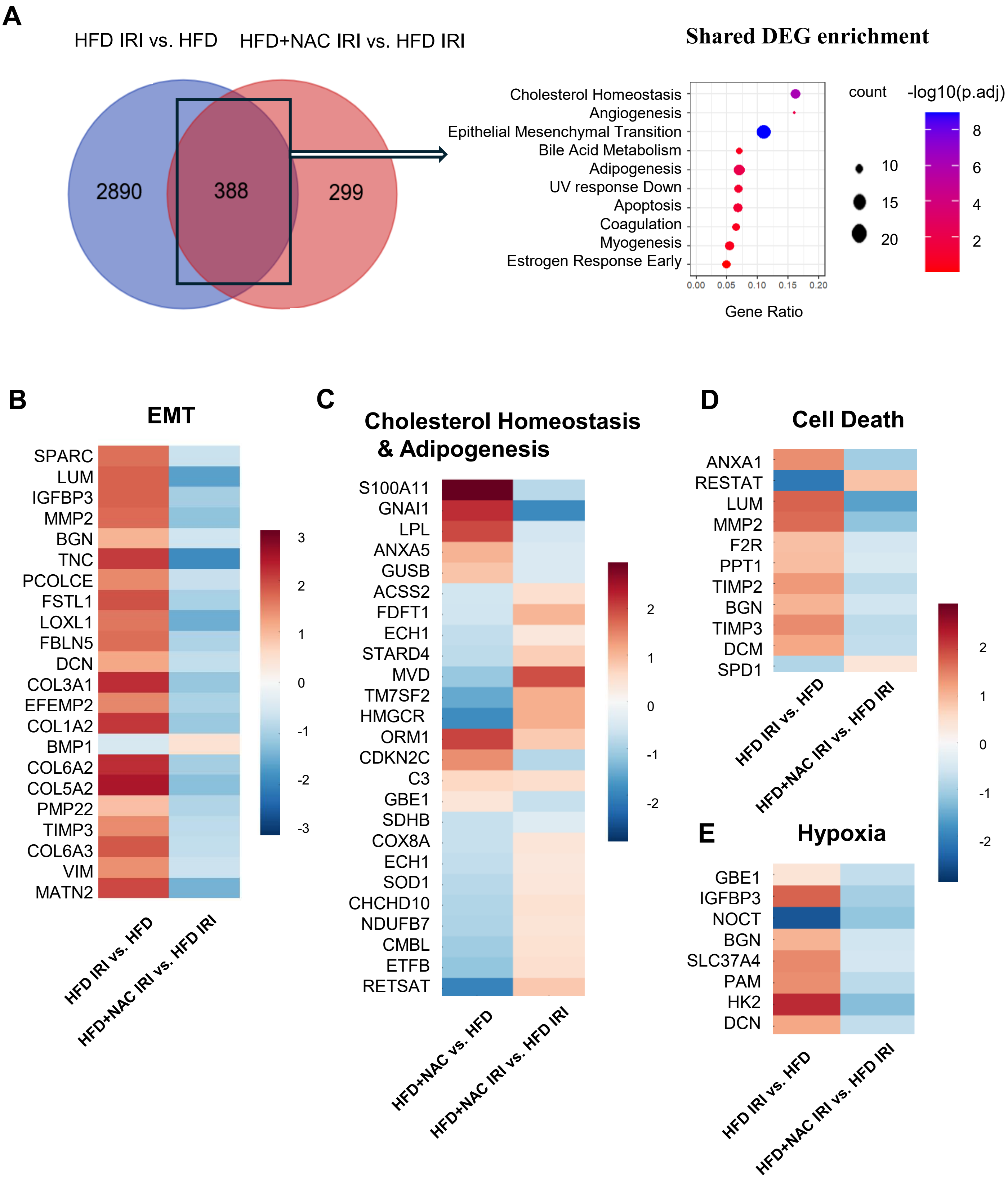

Supplementary Figure S5

A

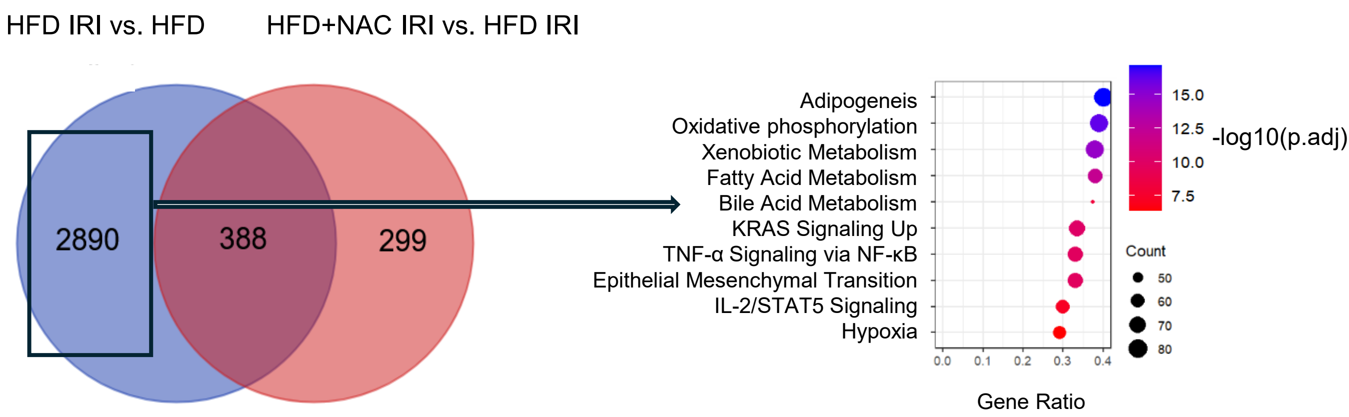

B

Pathways	Genes
Adipogenesis	COX7B;SLC27A1;MTCH2;PHLDB1;ACAA2;SOWAHC;COX6A1;ACADL;CPT2;ALDH2;NKIRAS1;DBT;ENPP2;ME1;PIM3;CD36;ACADM;HADH;ACADS;PGM1;HIBCH;BCKDHA;PHYH;DGAT1;IFNGR1;GPX3;MCCC1;LIFR;SLC5A6;POR;ARL4A;TST;COL4A1;ACOX1;CAT;UQCRC1;NDUFS3;SPARCL1;SUCLG1;PLIN2;SLC25A10;ANGPTL4;DLD;MGLL;ECHS1;PFKFB3;LAMA4;ATL2;AK2;UQCR11;MRPL15;UQCR10;TOB1;AGPAT3;SULT1A1;GRPEL1;DDT;UCP2;UBC;DNAJB9;APOE;DECR1;CAVIN1;NDUFA5;FZD4;GADD45A;CAVIN2;EPHX2;CIDEA;VEGFB;COQ9;FAH;DNAJC15;PEX14;QDPR;COQ3;FABP4;REEP5;UQCRQ;NDUFAB1;ACO2
Oxidative Phosphorylation	COX7B;ACAA2;ALAS1;ECI1;ETFA;MRPL34;COX6A1;PHB2;MPC1;IDH3B;TIMM17A;UQCRFS1;ACADM;BCKDHA;PHYH;NDUFC1;SDHA;COX6B1;HADHB;SUPV3L1;HADHA;OXA1L;POR;BDH2;NDUFS8;NDUFS7;NDUFS6;FDX1;NDUFS4;UQCRC1;NDUFS3;SUCLG1;SLC25A12;DLD;SLC25A4;NDUFB8;ECHS1;OAT;MAOB;UQCRB;NDUFB6;NDUFB5;TIMM9;NDUFB3;ETFDH;DLST;UQCR11;COX7A2;MRPL15;UQCR10;TIMM10;PDHB;COX5A;HSD17B10;UQCRH;ACAT1;GRPEL1;SLC25A20;DECR1;HSPA9;TIMM8B;CYB5A;SURF1;NDUFA7;NDUFA6;MDH1;NDUFA5;NDUFA4;GOT2;NDUFA1;COX6C;ACADSB;LRPPRC;ALDH6A1;SUCLA2;UQCRQ;NDUFAB1;CYCS;ACO2
Xenobiotic Metabolism	ALAS1;PROS1;TAT;AQP9;ATP2A2;TDO2;ALDH2;HNF4A;KYNU;CD36;HGFAC;MBL2;MCCC2;IGFBP1;ABCC3;HES6;GABARAPL1;G6PC;ABCC2;GSTO1;ARG1;ELOVL5;IGFBP4;DCXR;SLC6A12;PTGR1;PGD;UPB1;TNFRSF1A;CYP27A1;UGDH;POR;RBP4;MTHFD1;NPC1;ACOX1;CAT;IRF8;ALDH9A1;AHCY;SHMT2;SAR1B;SLC22A1;ETFDH;GSTT2;ABHD6;NDRG2;BPHL;PAPSS2;HSD11B1;PGRMC1;SERTAD1;DDT;TTPA;HSD17B2;HMOX1;APOE;CCL25;CYB5A;TGFB2;SLC12A4;PMM1;GCH1;SMOX;TMEM176B;PTGES3;FAH;GNMT;FABP1;GCLC;VNN1;GSTA3;ID2;CYP1A2;ACO2;LPIN2
Fatty Acid Metabolism	ACAA2;ACSM3;ECI1;ECI2;CPOX;MCEE;LGALS1;TDO2;ACADL;CPT2;AUH;ME1;IDH3B;MLYCD;ACADM;CD36;HADH;ACADS;HIBCH;YWHAH;ACOT8;GABARAPL1;HSP90AA1;HSDL2;ELOVL5;BCKDHB;SDHA;HADHB;UGDH;ACOX1;GPD1;SUCLG1;DLD;MGLL;ALDH9A1;GCDH;SLC22A5;ECHS1;HPGD;RAP1GDS1;ETFDH;HSD17B4;DLST;PDHB;HSD17B11;HSD17B10;BPHL;HMGCL;HSPH1;RDH16;HAO2;DECR1;MDH1;AADAT;CIDEA;FABP1;GSTZ1;FABP2;VNN1;SUCLA2;ACO2
KRAS Signaling Up	PIGR;PLVAP;FLT4;ITGB2;F13A1;TNFAIP3;PLAT;IKZF1;PRDM1;ETS1;CTSS;RBM4;ADGRA2;PLAU;C3AR1;SCN1B;CMKLR1;ST6GAL1;GUCY1A1;FCER1G;ARG1;VWA5A;EMP1;NGF;TNFRSF1B;EREG;ALDH1A3;RBP4;GPRC5B;MYCN;PECAM1;SPARCL1;IRF8;ADAM8;ANGPTL4;TRIB1;DOCK2;ENG;PPP1R15A;GYPC;CFH;RGS16;TFPI;CBL;CSF2RA;HDAC9;HSD11B1;RELN;GPNMB;TSPAN7;EPB41L3;SPP1;CBR4;MAP3K1;PTCD2;TMEM176B;IL10RA;TMEM176A;CIDEA;LAPTM5;GADD45G;DCBLD2;YRDC;MAFB;DNMBP;ID2;ADGRL4
TNF-α Signaling via NF-κB	BTG3;BTG2;B4GALT1;PLEK;TNFAIP2;TNFAIP3;IRS2;CXCL1;LITAF;SAT1;ICAM1;ZFP36;PLAU;KYNU;PMEPA1;IER5;IER2;IER3;TGIF1;DUSP1;FOS;RHOB;IRF1;SIK1;TRIB1;PLPP3;SGK1;IL6ST;CD44;TLR2;PPP1R15A;CEBPB;PFKFB3;SDC4;CEBPD;FUT4;SNN;NFIL3;DNAJB4;HES1;MSC;MCL1;EGR1;JUN;EGR2;JAG1;GADD45B;GCH1;LAMB3;GADD45A;PLK2;SOD2;KLF2;EIF1;VEGFA;NFKBIA;PER1;NR4A1;YRDC;KLF6;TNIP1;ID2;CLCF1;MAFF;BCL3;NFKBIE
Epithelial Mesenchymal Transition	ECM1;SERPINE2;ITGB5;WIPF1;ITGB3;COL12A1;TNFAIP3;PLOD2;CXCL1;LAMC1;SAT1;LOXL2;RGS4;GJA1;LGALS1;BASP1;PMEPA1;ITGAV;TIMP1;COLGALT1;TGM2;PDGFRB;POSTN;TNFRSF12A;IGFBP4;TPM2;TPM1;WNT5A;IGFBP2;APLP1;RHOB;TGFBR3;VCAN;COL4A2;COL4A1;PPIB;CD44;PFN2;LRRC15;SDC4;LAMA1;LAMA3;CAPG;TNFRSF11B;THBS2;NID2;FBLN2;THBS1;SERPINH1;SPP1;FLNA;MEST;JUN;VCAM1;TGFB1;GADD45B;GADD45A;FZD8;VEGFA;DAB2;SLC6A8;COL5A1;ID2;CALU;TGFBI;FBN1
Bile Acid Metabolism	PECR;ABCD3;AQP9;AKR1D1;HSD17B4;HSD17B6;HSD17B11;CYP7A1;FADS2;TTR;FDXR;LONP2;MLYCD;FADS1;PEX16;ABCG8;GSTK1;PHYH;PEX19;EPHX2;DIO1;ABCA9;PIPOX;APOA1;BBOX1;CYP7B1;CYP8B1;GNMT;CYP27A1;CYP39A1;SULT1B1;AMACR;NPC1;PEX6;PXMP2;CAT;PEX11G;AGXT;SLC29A1;SLC27A5;OPTN;ALDH9A1
IL-2/STAT5 Signaling	CDKN1C;ECM1;GUCY1B1;PTH1R;AHR;IKZF2;IGF1R;SPRED2;CCND3;ALCAM;NFKBIZ;TNFSF10;SH3BGRL2;ITGAV;WLS;TGM2;GABARAPL1;BATF3;IFNGR1;GSTO1;S100A1;ANXA4;APLP1;EMP1;RHOH;FAM126B;TNFRSF1B;CKAP4;RHOB;PLSCR1;ARL4A;CDCP1;CCNE1;RRAGD;IRF8;TLR7;CD48;PLIN2;ITGA6;CD44;TNFRSF21;AHCY;AHNAK;SERPINC1;RGS16;CAPG;NFIL3;PLAGL1;SPP1;GADD45B;IL10RA;FAH;HOPX;TTC39B;KLF6;AMACR;MYO1C;MAFF;F2RL2;CDC42SE2
Hypoxia	CDKN1C;ERRFI1;TES;TNFAIP3;IRS2;VLDLR;ETS1;HK1;ZFP36;CSRP2;FAM162A;STBD1;KIF5A;LARGE1;PPFIA4;PGM1;PDK1;IER3;TGM2;IGFBP1;TPD52;DUSP1;FOS;JMJD6;DDIT4;RRAGD;PKP1;MYH9;PLIN2;ANGPTL4;ALDOB;CHST2;PPP1R15A;PFKFB3;SDC4;SDC3;PDGFB;FOXO3;SELENBP1;NFIL3;STC2;GPC3;HMOX1;VHL;PCK1;JUN;MAP3K1;PKLR;CAVIN1;HSPA5;CP;VEGFA;EXT1;KLF6;NAGK;COL5A1;MAFF;TGFBI

Supplementary Figure S6

A

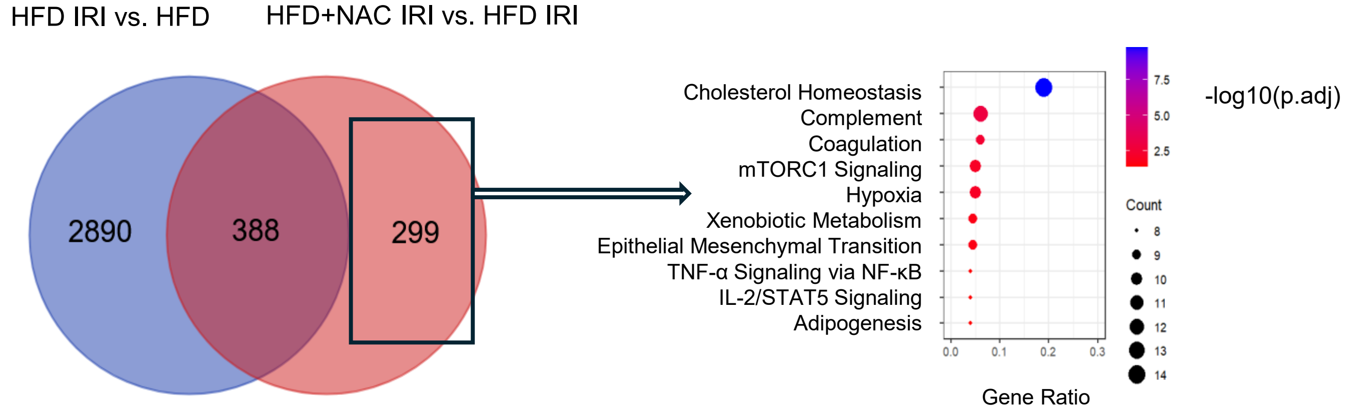

B

Pathways	Genes
Cholesterol Homeostasis	FDPS;MVK;HMGCS1;TMEM97;AVPR1A;LSS;SREBF2;NSDHL;ATXN2;FABP5; PMVK;MAL2;ALDOC;DHCR7
Complement	CDA;LRP1;C9;GPD2;SERPINE1;FN1;PIM1;SERPING1;PCSK9;ANG;ITIH1;CFB
Coagulation	THBD;LRP1;C9;SERPINE1;FN1;SERPING1;ANG;ITIH1;CFB
mTORC1 Signaling	ACTR3;ACTR2;RRM2;HMGCS1;RDH11;INSIG1;ELOVL6;TMEM97;ENO1;DHCR7
Hypoxia	EFNA1;UGP2;TGFB3;GAA;SERPINE1;PIM1;ACKR3;ALDOC;ENO1;EGFR
Xenobiotic Metabolism	CRP;CDA;VTN;TKFC;SERPINE1;FAS;TMEM97;ITIH1;CFB
Epithelial Mesenchymal Transition	COL1A1;NT5E;LRP1;SERPINE1;FN1;FAS;QSOX1;INHBA;FMOD
TNF-α Signaling via NF-κB	EFNA1;KDM6B;CCND1;BCL6;SERPINE1;ACKR3;INHBA;JUNB
IL-2/STAT5 Signaling	SOCS2;NRP1;NT5E;CISH;PIM1;LRRC8C;BCL2L1;PLEC
Adipogenesis	LIPE;RNF11;BCL6;GPAT4;GPD2;ELMOD3;ELOVL6;DHCR7

Supplementary Figure S7

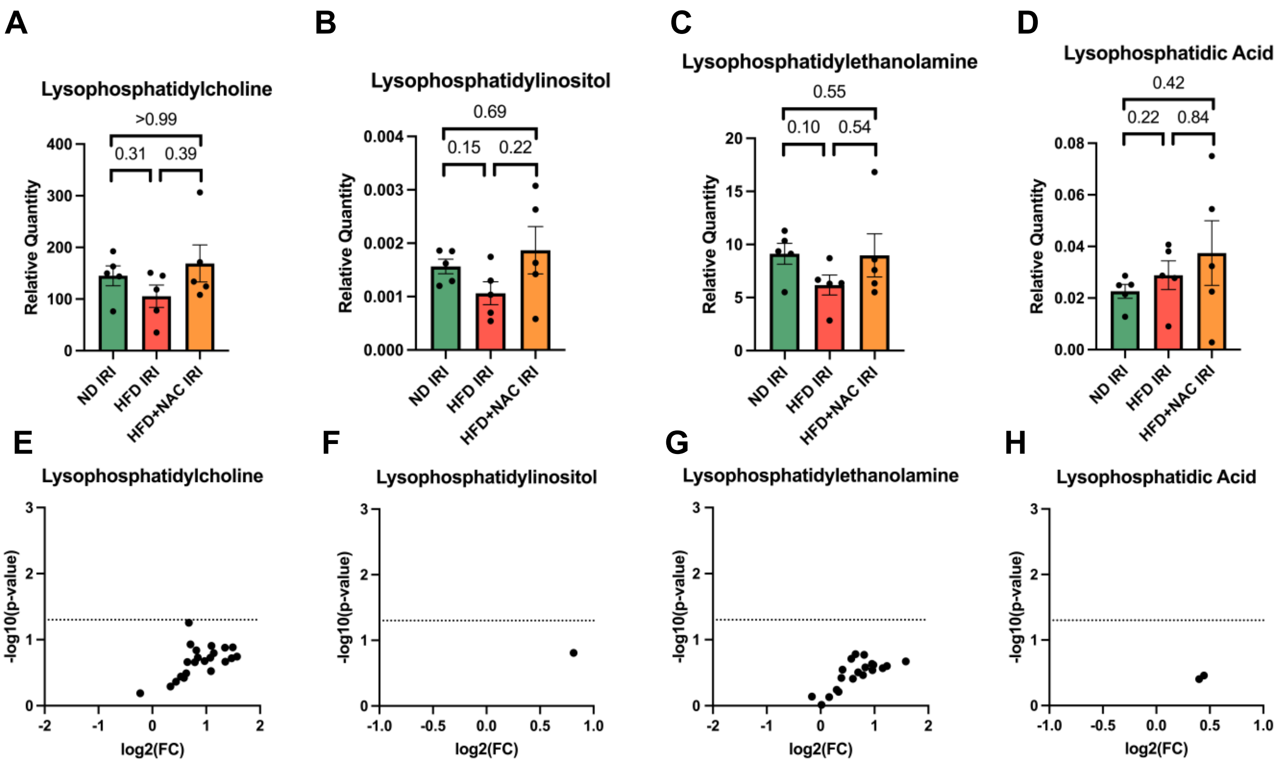

Supplementary Figure S8

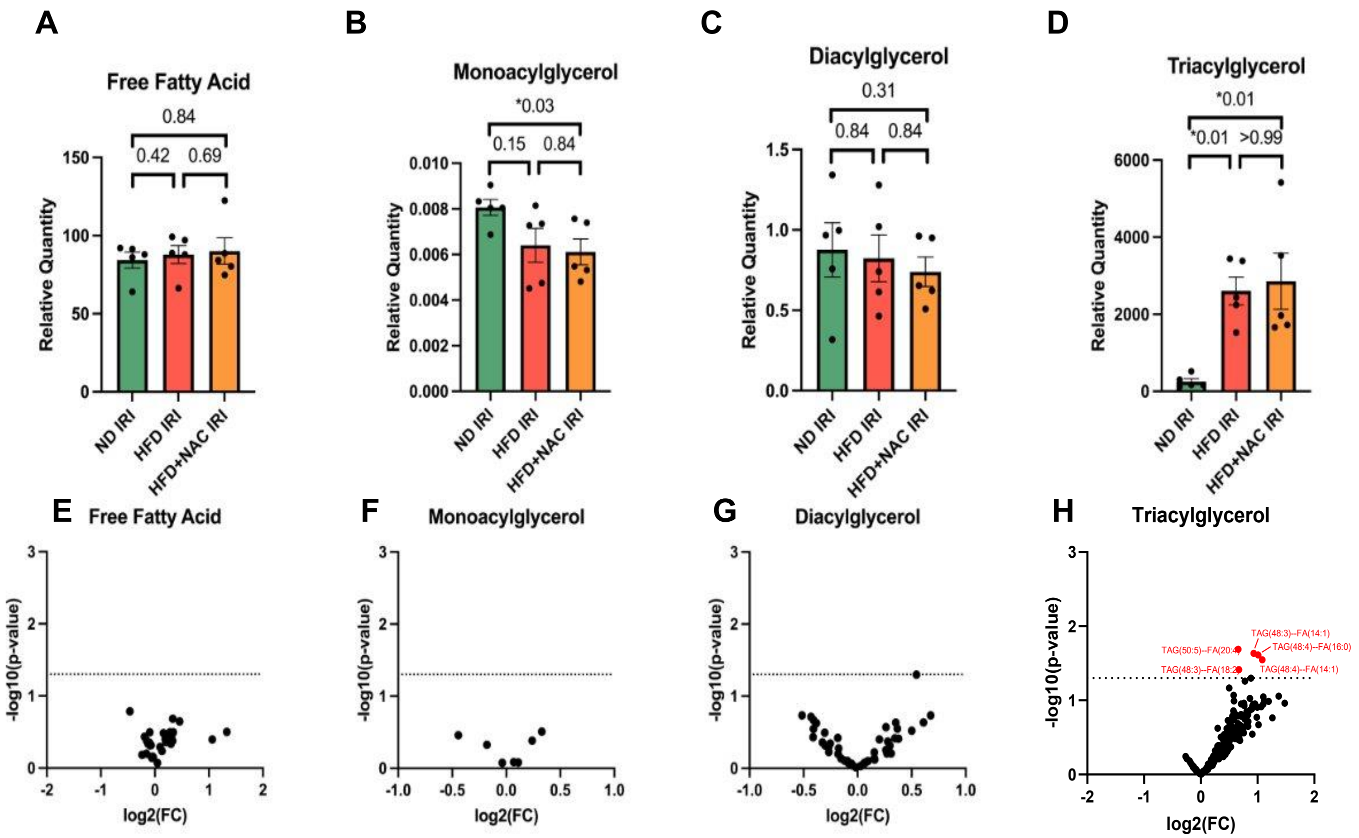

Supplementary Figure S9

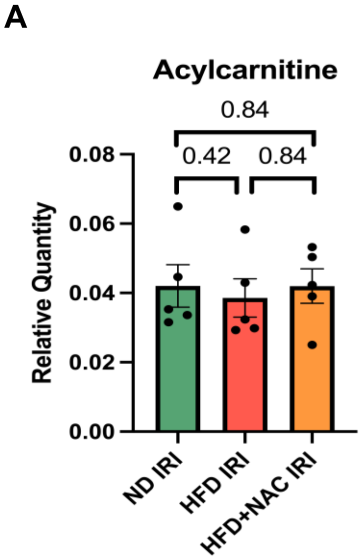

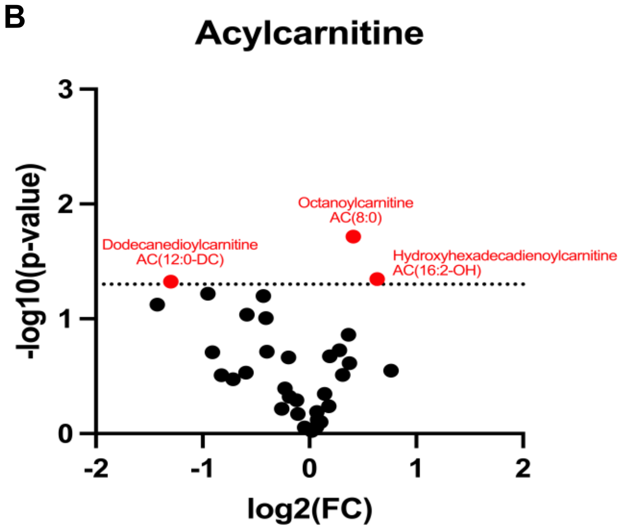

Supplementary Figure S10

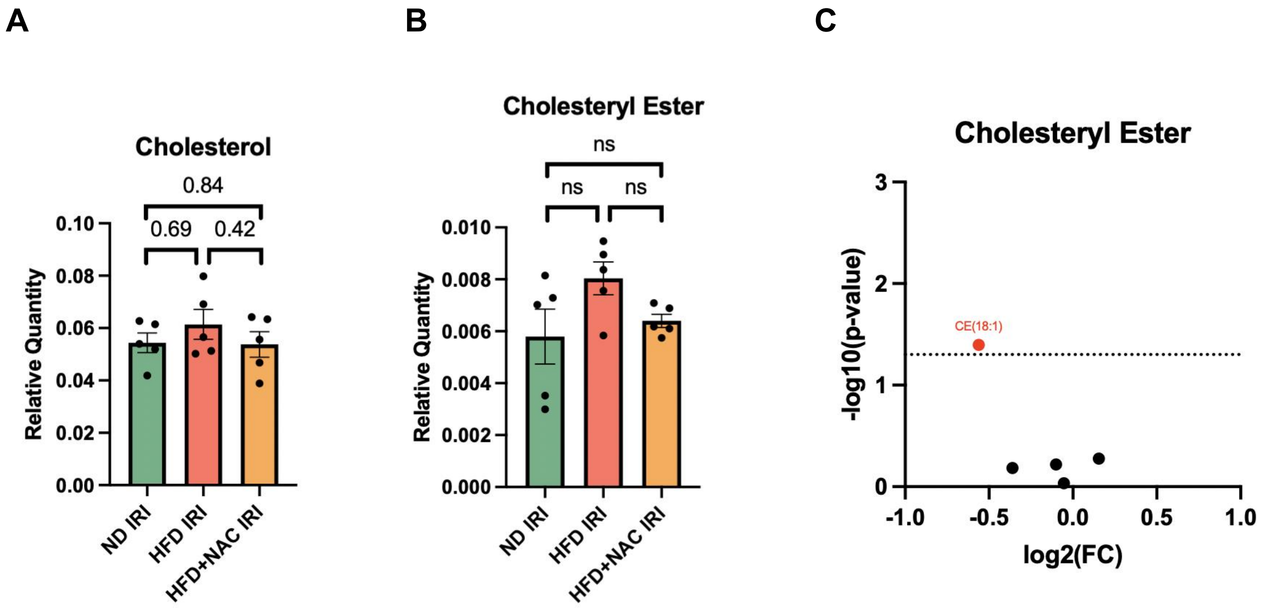

Supplementary Figure S11

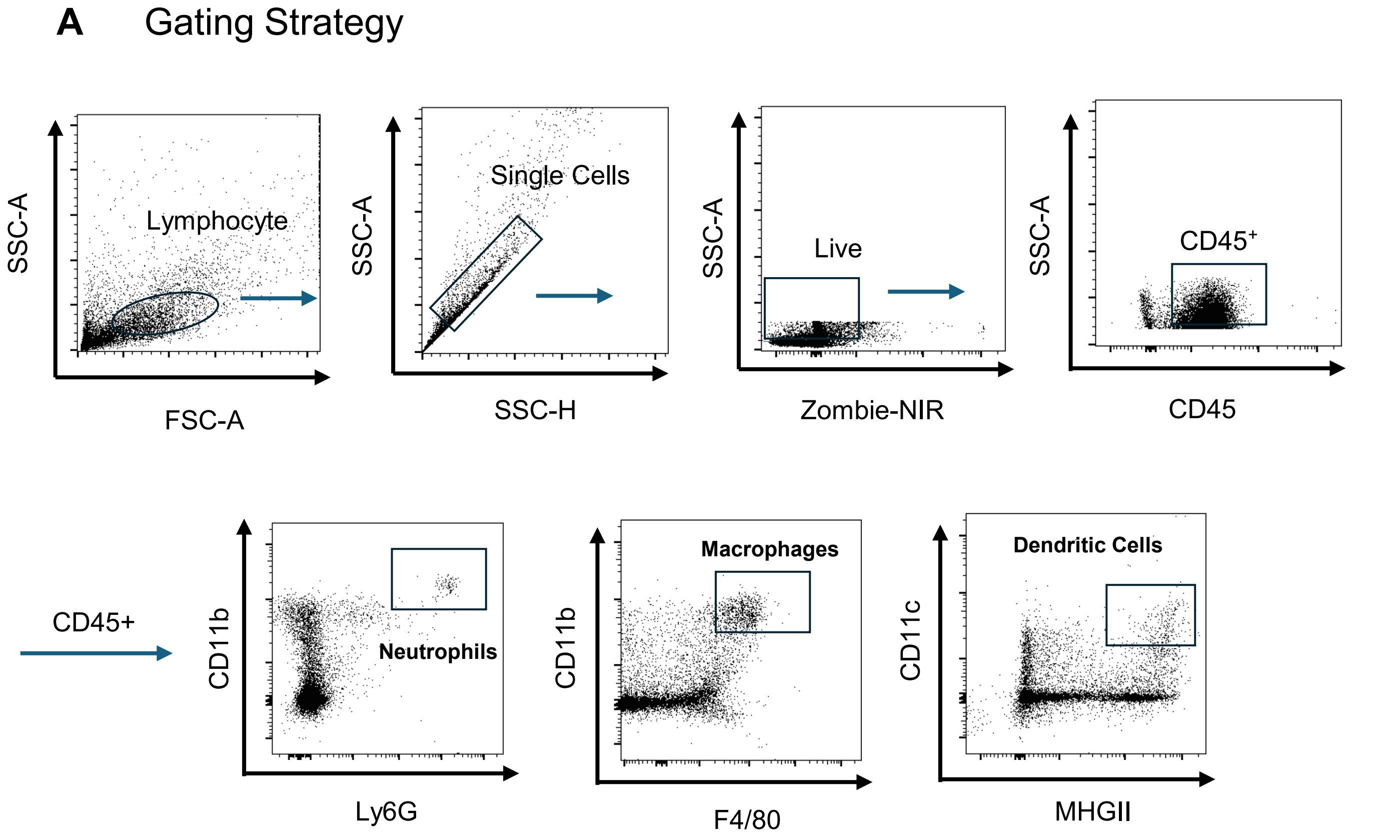

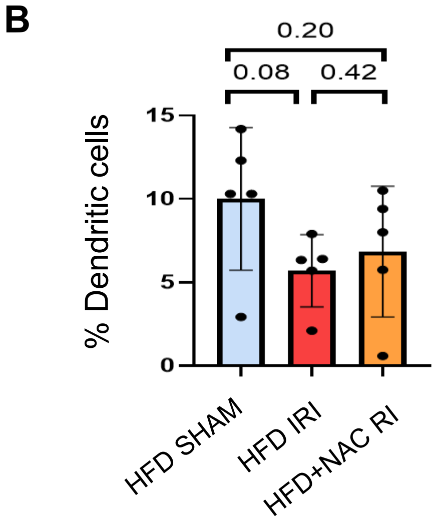

Supplementary Table S1

Trend	Lipid species
Significantly Elevated Lipids	CER(14:0), CER(16:0), DCER(26:0), HCER(14:0), HCER(18:0), HCER(18:1), HCER(20:0), HCER(20:1), HCER(22:0), HCER(22:1), Hydroxyhexadecadienoylcarnitine_AC(16:2-OH), Octanoylcarnitine_AC(8:0), PC(12:0/18:1), PC(14:0/16:1), PC(14:0/20:1), PC(14:0/20:2), PC(15:0/18:1), PC(15:0/18:2), PC(16:0/14:0), PC(16:0/16:1), PC(16:0/18:1), PC(16:0/18:3), PC(16:0/20:4), PC(16:0/22:4), PC(16:0/22:5), PC(17:0/18:1), PC(18:0/12:0), PC(18:0/16:1), PC(18:0/18:0), PC(18:0/20:1), PC(18:0/20:4), PC(18:0/22:4), PC(18:1/16:1), PC(18:1/20:1), PC(18:1/20:2), PC(18:1/22:4), PC(18:2/16:1), PC(18:2/22:5), PC(18:2/22:6), PC(O-34:0)_184, PE(14:0/22:6), PE(16:0/18:2), PE(16:0/20:5), PE(16:0/22:4), PE(16:0/22:5), PE(18:0/16:1), PE(18:0/22:4), PE(18:1/20:1), PE(18:1/20:2), PE(18:1/20:3), PE(18:1/22:4), PE(18:1/22:5), PE(18:2/16:1), PE(18:2/20:1), PE(18:2/20:2), PE(O-16:0/18:1), PE(O-18:0/20:5), PI(16:1/18:1), PI(18:0/22:4), PS(16:1/18:0), SM(24:0), SM(24:1), SM(d18:1/14:1), SM(d18:1/16:0), SM(d18:1/16:1), SM(d18:1/17:0), SM(d18:1/20:0), SM(d18:1/20:1), SM(d18:1/20:2), SM(d18:1/22:1), SM(d18:1/22:2), SM(d18:1/22:3), TAG48:3-FA14:1, TAG48:3-FA18:2, TAG48:4-FA14:1, TAG48:4-FA16:0, TAG50:5-FA20:4
Lipids with No Significant Difference	Carboxytridecenoylcarnitine_AC(14:1-DC), CE(18:2), CE(20:5), CE(22:6), CE(24:0), CER(18:0), CER(18:1), CER(20:0), CER(20:1), CER(22:0), CER(22:1), CER(24:0), CER(24:1), CER(26:0), CER(26:1), Cholesterol, DAG(12:0/18:0), DAG(12:0/18:1), DAG(14:0/18:1), DAG(14:0/18:2), DAG(14:0/20:4), DAG(14:0/22:6), DAG(14:1/16:0), DAG(14:1/18:1), DAG(15:0/18:2), DAG(16:0/16:0), DAG(16:0/16:1), DAG(16:0/18:0), DAG(16:0/18:1), DAG(16:0/18:2), DAG(16:0/18:3), DAG(16:0/20:3), DAG(16:0/20:4), DAG(16:0/20:5), DAG(16:0/22:5), DAG(16:0/22:6), DAG(16:1/16:1), DAG(16:1/18:0), DAG(16:1/18:1), DAG(16:1/18:2), DAG(16:1/18:3), DAG(16:1/20:2), DAG(16:1/20:4), DAG(16:1/22:6), DAG(18:0/18:1), DAG(18:0/18:2), DAG(18:0/18:3), DAG(18:0/22:6), DAG(18:1/18:1), DAG(18:1/18:2), DAG(18:1/20:1), DAG(18:1/20:2), DAG(18:1/20:3), DAG(18:1/20:4), DAG(18:1/20:5), DAG(18:1/22:4), DAG(18:1/22:5), DAG(18:1/22:6), DAG(18:2/18:3), DAG(18:2/20:3), DAG(18:2/20:4), DAG(18:2/20:5), DAG(18:2/22:4), DAG(18:2/22:5), DAG(18:2/22:6), DCER(16:0), DCER(18:0), DCER(20:0), DCER(22:0), DCER(22:1), DCER(24:0), DCER(24:1), Decadienoylcarnitine_AC(10:2), Decenoylcarnitine_AC(10:1), Decenoylcarnitine_C10:1, Dimethylarginine, Dimethylnonanoylcarnitine_AC(11:0), Dodecanoylcarnitine_AC(12:0), Dodecenoylcarnitine_AC(12:1), FFA(11:0), FFA(12:0), FFA(14:0), FFA(14:1), FFA(15:0), FFA(16:0), FFA(16:1), FFA(17:0), FFA(18:0), FFA(18:1), FFA(18:2), FFA(18:3), FFA(18:4), FFA(20:0), FFA(20:1), FFA(20:2), FFA(20:3), FFA(20:4), FFA(20:5), FFA(22:0), FFA(22:1), FFA(22:2), FFA(22:4), FFA(22:4)_1, FFA(22:5), FFA(22:6), FFA(24:0), FFA(24:1), FFA(9:0), HCER(16:0), HCER(24:0), HCER(24:1), HCER(26:0), HCER(26:1), Heptadecanoylcarnitine_AC(17:0), Heptanoylcarnitine_AC(7:0), Hexadecadienoylcarnitine_AC(16:2), Hexadecadienyl-L-carnitine_C16:2, Hexadecanoylcarnitine_AC(16:0), Hexadecenoylcarnitine_AC(16:1), Hydroxyhexadecenoylcarnitine_AC(16:1-OH), Hydroxymyristoylcarnitine_AC(14:0-OH), Hydroxyoctadecenoylcarnitine_AC(18:1-OH), Hydroxyoctadecenoylcarnitine_C18:1-OH, Hydroxyoctenoylcarnitine_AC(8:1-OH), Hydroxytetradecenoylcarnitine_AC(14:1-OH), IS_FFA_1, LCER(16:0), LCER(18:0), LCER(20:0), LCER(22:0), LCER(22:1), LCER(24:0), LCER(24:1), LPA(16:0), LPA(16:1), LPC(14:0), LPC(14:1), LPC(15:0), LPC(16:0), LPC(16:1), LPC(17:0), LPC(18:0), LPC(18:1), LPC(18:2), LPC(18:3), LPC(18:4), LPC(20:0), LPC(20:1), LPC(20:2), LPC(20:3), LPC(20:4), LPC(20:5), LPC(22:0), LPC(22:1), LPC(22:5), LPC(22:6), LPC(24:0), LPC(24:1), LPE(15:0), LPE(16:0), LPE(16:1), LPE(17:0), LPE(18:0), LPE(18:1), LPE(18:2), LPE(18:3), LPE(20:0), LPE(20:1), LPE(20:2), LPE(20:3), LPE(20:4), LPE(22:0), LPE(22:1), LPE(22:4), LPE(22:5), LPE(22:6), LPE(24:0), LPE(24:1), LPI(16:1), MAG(16:0), MAG(16:1), MAG(18:1), MAG(18:2), MAG(20:3), MAG(20:4), MAG(22:5), Nonaylcarnitine_AC(9:0), Octadecanoylcarnitine_AC(18:0), Octadecenoylcarnitine_AC(18:1), Octenoylcarnitine_AC(8:1), O-nonanoyl-L-carnitine_C9, PA(16:0/18:0), PA(16:0/18:2), PA(18:0/18:2), PA(18:0/20:3), PA(18:1/18:2), PA(22:0/22:6), PC(12:0/16:1), PC(12:0/18:2), PC(14:0/14:0), PC(14:0/18:1), PC(14:0/18:2), PC(14:0/20:3), PC(14:0/20:4), PC(14:0/22:6), PC(15:0/16:1), PC(16:0/12:0), PC(16:0/16:0), PC(16:0/18:0), PC(16:0/18:2), PC(16:0/20:1), PC(16:0/20:2), PC(16:0/20:3), PC(16:0/20:5), PC(16:0/22:6), PC(17:0/16:1), PC(17:0/18:2), PC(17:0/20:3), PC(17:0/20:4), PC(17:0/20:5), PC(18:0/18:1), PC(18:0/18:2), PC(18:0/18:3), PC(18:0/20:0), PC(18:0/20:2), PC(18:0/20:3), PC(18:0/22:5), PC(18:1/18:1), PC(18:1/18:2), PC(18:1/18:3), PC(18:1/20:3), PC(18:1/22:5), PC(18:2/18:2), PC(18:2/18:3), PC(18:2/20:2), PC(18:2/20:3), PC(18:2/20:4), PC(18:2/20:5), PC(20:0/18:1), PC(20:0/18:2), PC(20:0/20:3), PC(20:0/20:4), PC(20:0/22:4), PC(20:0/22:5), PC(20:0/22:6), PC(32:0)_184, PC(34:4)_184, PC(36:6)_184, PC(38:0)_184, PC(38:3)_184, PC(38:6)_184, PC(40:1)_184, PC(40:5)_184, PC(O-36:4)_184, PC(O-42:1)_184, PE(14:0/16:1), PE(14:0/18:1), PE(14:0/18:2), PE(14:0/20:4), PE(14:0/22:2), PE(14:0/22:5), PE(15:0/20:2), PE(16:0/14:0), PE(16:0/14:1), PE(16:0/16:0), PE(16:0/16:1), PE(16:0/18:1), PE(16:0/18:3), PE(16:0/20:1), PE(16:0/20:2), PE(16:0/20:3), PE(16:0/20:4), PE(16:0/22:6), PE(17:0/18:1), PE(17:0/18:2), PE(17:0/20:4), PE(17:0/22:5), PE(18:0/15:0), PE(18:0/16:0), PE(18:0/18:0), PE(18:0/18:1), PE(18:0/18:2), PE(18:0/18:3), PE(18:0/20:1), PE(18:0/20:2), PE(18:0/20:3), PE(18:0/20:4), PE(18:0/20:5), PE(18:0/22:5), PE(18:0/22:6), PE(18:1/14:1), PE(18:1/16:1), PE(18:1/18:1), PE(18:1/18:2), PE(18:1/18:3), PE(18:1/20:4), PE(18:1/20:5), PE(18:1/22:6), PE(18:2/14:1), PE(18:2/18:2), PE(18:2/18:3), PE(O-16:0/16:0), PE(O-16:0/16:1), PE(O-16:0/18:2), PE(O-16:0/20:3), PE(O-16:0/20:4), PE(O-16:0/20:5), PE(O-16:0/22:4), PE(O-16:0/22:5), PE(O-16:0/22:6), PE(O-18:0/16:0), PE(O-18:0/16:1), PE(O-18:0/18:1), PE(O-18:0/20:3), PE(O-18:0/20:4), PE(O-18:0/22:4), PE(O-18:0/22:5), PE(O-18:0/22:6), PE(P-14:0/18:1), PE(P-16:0/16:0), PE(P-16:0/18:1), PE(P-16:0/18:2), PE(P-16:0/20:1), PE(P-16:0/20:3), PE(P-16:0/20:4), PE(P-16:0/20:5), PE(P-16:0/22:4), PE(P-16:0/22:5), PE(P-16:0/22:6), PE(P-18:0/16:0), PE(P-18:0/16:1), PE(P-18:0/18:1), PE(P-18:0/18:2), PE(P-18:0/20:1), PE(P-18:0/20:2), PE(P-18:0/20:3), PE(P-18:0/20:4), PE(P-18:0/20:5), PE(P-18:0/22:4), PE(P-18:0/22:5), PE(P-18:0/22:6), PE(P-18:1/16:0), PE(P-18:1/18:2), PE(P-18:1/20:3), PE(P-18:1/20:4), PE(P-18:1/20:5), PE(P-18:1/22:4), PE(P-18:1/22:5), PE(P-18:1/22:6), PE(P-18:2/20:4), PE(P-18:2/22:6),

Continued

Trend	Lipid species
Lipids with No Significant Difference	PG(14:0/16:0), PG(16:0/16:0), PG(16:0/16:1), PG(16:0/18:0), PG(16:0/18:1), PG(16:0/18:2), PG(16:0/20:2), PG(16:0/20:3), PG(16:1/16:1), PG(16:1/18:0), PG(16:1/18:1), PG(16:1/18:2), PG(18:0/18:0), PG(18:0/18:1), PG(18:0/18:2), PG(18:0/20:4), PG(18:1/18:1), PG(18:1/18:2), PG(18:1/18:3), PG(18:2/18:2), PI(14:0/18:0), PI(14:0/18:1), PI(16:0/16:0), PI(16:0/16:1), PI(16:0/18:0), PI(16:0/18:1), PI(16:0/18:2), PI(16:0/20:0), PI(16:0/20:3), PI(16:0/20:4), PI(16:1/18:0), PI(16:1/18:2), PI(18:0/18:0), PI(18:0/18:1), PI(18:0/18:2), PI(18:0/18:3), PI(18:0/20:2), PI(18:0/20:3), PI(18:0/20:4), PI(18:0/20:5), PI(18:1/18:1), PI(18:1/18:2), PI(18:1/18:3), PI(18:1/20:3), PI(18:2/18:2), PI(18:2/20:1), PI(20:0/20:3), PI(20:0/20:4), Pimeloylcarnitine_AC(7:0-DC), Propionylcarnitine_C3, PS(16:0/16:0), PS(16:0/18:0), PS(16:0/18:1), PS(16:0/18:2), PS(18:0/18:0), PS(18:0/18:1), PS(18:0/18:2), PS(18:1/18:1), PS(18:1/18:2), SM(26:0), SM(26:1), SM(d18:1/12:0), SM(d18:1/14:0), SM(d18:1/18:0), SM(d18:1/18:1), SM(d18:1/18:2), SM(d18:1/18:3), SM(d18:1/18:4), SM(d18:1/20:3), SM(d18:1/20:4), SM(d18:1/20:5), SM(d18:1/22:0), SM(d18:1/22:4), SM(d18:1/22:5), SM(d18:1/22:6), Succinylcarnitine/3 hydroxyisovalerylcarnitine_C5-OH(C3-DC-M), TAG48:0-FA14:0, TAG48:0-FA16:0, TAG48:0-FA18:0, TAG48:1-FA14:0, TAG48:1-FA14:1, TAG48:1-FA16:0, TAG48:1-FA16:1, TAG48:1-FA18:0, TAG48:1-FA18:1, TAG48:2-FA14:0, TAG48:2-FA14:1, TAG48:2-FA16:0, TAG48:2-FA16:1, TAG48:2-FA18:1, TAG48:2-FA18:2, TAG48:3-FA12:0, TAG48:3-FA14:0, TAG48:3-FA16:0, TAG48:3-FA16:1, TAG48:3-FA18:1, TAG48:4-FA14:0, TAG48:4-FA16:1, TAG48:4-FA18:1, TAG48:4-FA18:2, TAG48:4-FA18:3, TAG50:0-FA14:0, TAG50:0-FA16:0, TAG50:0-FA18:0, TAG50:1-FA14:0, TAG50:1-FA16:0, TAG50:1-FA16:1, TAG50:1-FA18:0, TAG50:1-FA18:1, TAG50:2-FA14:0, TAG50:2-FA14:1, TAG50:2-FA16:0, TAG50:2-FA16:1, TAG50:2-FA18:0, TAG50:2-FA18:1, TAG50:2-FA18:2, TAG50:3-FA14:0, TAG50:3-FA14:1, TAG50:3-FA16:0, TAG50:3-FA16:1, TAG50:3-FA18:0, TAG50:3-FA18:1, TAG50:3-FA18:2, TAG50:3-FA18:3, TAG50:3-FA20:3, TAG50:4-FA14:0, TAG50:4-FA14:1, TAG50:4-FA16:0, TAG50:4-FA16:1, TAG50:4-FA18:1, TAG50:4-FA18:2, TAG50:4-FA18:3, TAG50:4-FA20:4, TAG50:5-FA14:0, TAG50:5-FA16:0, TAG50:5-FA16:1, TAG50:5-FA18:1, TAG50:5-FA18:2, TAG50:5-FA18:3, TAG50:5-FA20:5, TAG52:0-FA16:0, TAG52:0-FA18:0, TAG52:0-FA20:0, TAG52:1-FA16:0, TAG52:1-FA16:1, TAG52:1-FA18:0, TAG52:1-FA18:1, TAG52:1-FA20:0, TAG52:1-FA20:1, TAG52:2-FA14:0, TAG52:2-FA16:0, TAG52:2-FA16:1, TAG52:2-FA18:0, TAG52:2-FA18:1, TAG52:2-FA18:2, TAG52:2-FA20:0, TAG52:2-FA20:1, TAG52:2-FA20:2, TAG52:3-FA14:0, TAG52:3-FA16:0, TAG52:3-FA16:1, TAG52:3-FA18:0, TAG52:3-FA18:1, TAG52:3-FA18:2, TAG52:3-FA18:3, TAG52:3-FA20:0, TAG52:3-FA20:1, TAG52:3-FA20:2, TAG52:3-FA20:3, TAG52:3-FA22:1, TAG52:4-FA14:0, TAG52:4-FA16:0, TAG52:4-FA16:1, TAG52:4-FA18:0, TAG52:4-FA18:1, TAG52:4-FA18:2, TAG52:4-FA18:3, TAG52:4-FA20:0, TAG52:4-FA20:2, TAG52:4-FA20:3, TAG52:4-FA20:4, TAG52:4-FA22:1, TAG52:4-FA22:4, TAG52:5-FA14:0, TAG52:5-FA16:0, TAG52:5-FA16:1, TAG52:5-FA18:1, TAG52:5-FA18:2, TAG52:5-FA18:3, TAG52:5-FA20:3, TAG52:5-FA20:4, TAG52:5-FA20:5, TAG52:5-FA22:5, TAG52:6-FA14:0, TAG52:6-FA16:0, TAG52:6-FA16:1, TAG52:6-FA18:1, TAG52:6-FA18:2, TAG52:6-FA18:3, TAG52:6-FA20:4, TAG52:6-FA20:5, TAG52:6-FA22:6, TAG52:7-FA16:0, TAG52:7-FA20:5, TAG52:7-FA22:6, TAG52:8-FA16:1, TAG52:8-FA18:2, TAG54:0-FA16:0, TAG54:0-FA18:0, TAG54:1-FA16:0, TAG54:1-FA18:0, TAG54:1-FA18:1, TAG54:1-FA20:0, TAG54:1-FA20:1, TAG54:2-FA16:0, TAG54:2-FA18:0, TAG54:2-FA18:1, TAG54:2-FA18:2, TAG54:2-FA20:0, TAG54:2-FA20:1, TAG54:2-FA20:2, TAG54:3-FA16:0, TAG54:3-FA16:1, TAG54:3-FA18:0, TAG54:3-FA18:1, TAG54:3-FA18:2, TAG54:3-FA18:3, TAG54:3-FA20:1, TAG54:3-FA20:2, TAG54:3-FA20:3, TAG54:4-FA16:0, TAG54:4-FA16:1, TAG54:4-FA18:0, TAG54:4-FA18:1, TAG54:4-FA18:2, TAG54:4-FA18:3, TAG54:4-FA20:1, TAG54:4-FA20:2, TAG54:4-FA20:3, TAG54:4-FA20:4, TAG54:4-FA22:1, TAG54:4-FA22:4, TAG54:5-FA16:0, TAG54:5-FA16:1, TAG54:5-FA18:0, TAG54:5-FA18:1, TAG54:5-FA18:2, TAG54:5-FA18:3, TAG54:5-FA20:2, TAG54:5-FA20:3, TAG54:5-FA20:4, TAG54:5-FA20:5, TAG54:5-FA22:1, TAG54:5-FA22:4, TAG54:5-FA22:5, TAG54:6-FA16:0, TAG54:6-FA16:1, TAG54:6-FA18:1, TAG54:6-FA18:2, TAG54:6-FA18:3, TAG54:6-FA20:3, TAG54:6-FA20:4, TAG54:6-FA20:5, TAG54:6-FA22:5, TAG54:6-FA22:6, TAG54:7-FA16:1, TAG54:7-FA18:1, TAG54:7-FA18:2, TAG54:7-FA18:3, TAG54:7-FA20:4, TAG54:7-FA20:5, TAG54:7-FA22:5, TAG54:7-FA22:6, TAG54:8-FA18:2, TAG54:8-FA18:3, TAG54:8-FA20:4, TAG54:8-FA20:5, TAG54:8-FA22:6, TAG56:10-FA18:2, TAG56:1-FA16:0, TAG56:1-FA18:1, TAG56:2-FA16:0, TAG56:2-FA18:0, TAG56:2-FA20:0, TAG56:2-FA20:1, TAG56:3-FA16:0, TAG56:3-FA18:0, TAG56:3-FA18:1, TAG56:3-FA18:2, TAG56:3-FA20:0, TAG56:3-FA20:1, TAG56:3-FA20:2, TAG56:4-FA16:0, TAG56:4-FA18:0, TAG56:4-FA18:1, TAG56:4-FA18:2, TAG56:4-FA20:1, TAG56:4-FA20:2, TAG56:4-FA20:3, TAG56:4-FA20:4, TAG56:4-FA22:4, TAG56:5-FA16:0, TAG56:5-FA18:0, TAG56:5-FA18:1, TAG56:5-FA18:2, TAG56:5-FA20:1, TAG56:5-FA20:2, TAG56:5-FA20:3, TAG56:5-FA20:4, TAG56:5-FA22:4, TAG56:5-FA22:5, TAG56:6-FA16:0, TAG56:6-FA18:0, TAG56:6-FA18:1, TAG56:6-FA18:2, TAG56:6-FA18:3, TAG56:6-FA20:2, TAG56:6-FA20:3, TAG56:6-FA20:4, TAG56:6-FA20:5, TAG56:6-FA22:4, TAG56:6-FA22:5, TAG56:6-FA22:6, TAG56:7-FA16:0, TAG56:7-FA16:1, TAG56:7-FA18:0, TAG56:7-FA18:1, TAG56:7-FA18:2, TAG56:7-FA18:3, TAG56:7-FA20:3, TAG56:7-FA20:4, TAG56:7-FA20:5, TAG56:7-FA22:4, TAG56:7-FA22:5, TAG56:7-FA22:6, TAG56:8-FA16:0, TAG56:8-FA16:1, TAG56:8-FA18:1, TAG56:8-FA18:2, TAG56:8-FA18:3, TAG56:8-FA20:4, TAG56:8-FA20:5, TAG56:8-FA22:5, TAG56:8-FA22:6, TAG56:9-FA18:3, TAG56:9-FA20:4, TAG56:9-FA20:5, TAG56:9-FA22:6, Tetradecadienoylcarnitine_AC(14:2), Tetradecanoylcarnitine_AC(14:0), Tetradecenoylcarnitine_AC(14:1)
Significantly Decreased Lipids	CE(18:1), Dodecanedioylcarnitine_AC(12:0-DC)
